# Vapor-Phase Synthesis of Ti_2_CCl_2_ MXene

**DOI:** 10.1021/jacs.6c10774

**Published:** 2026-08-17

**Authors:** Hyunho Kim, Jongyoun Kim, Teng Zhang, Swarnendu Das, Yasunori Hioki, Eric A. Stach, Yury Gogotsi

**Affiliations:** † A. J. Drexel Nanomaterials Institute and Department of Materials Science and Engineering, 6527 Drexel University, Philadelphia, Pennsylvania 19104, United States; ‡ Department of Materials Science and Engineering, 6572 University of Pennsylvania, Philadelphia, Pennsylvania 19104, United States; § Murata Manufacturing Co., Ltd., 1-10-1 Higashikotari, Nagaokakyo-shi, Kyoto 617−8555, Japan; ∥ School of Advanced Materials Science and Engineering, Sungkyunkwan University, Seobu-Ro 2066, Jangan-Gu, Suwon-Si, Gyeonggi-Do 16419, Republic of Korea

## Abstract

The vapor-phase synthesis can produce 2D transition metal carbides and nitrides (MXenes) from abundant precursors, bypassing MAX phase synthesis. However, little progress was made after the initial report by Talapin’s group on the chemical vapor deposition of MXenes on titanium foil, as the growth mechanisms remained poorly understood. Here, a scalable vapor-phase synthesis of high-quality Ti_2_CCl_2_ MXene from Ti, TiCl_4_, and CH_4_ is demonstrated, and the key features of the growth mechanism are elucidated. Increasing the Ti surface area while confining the reaction volume enables vapor-phase formation of Ti_2_CCl_2_ on quartz substrates. Supersaturation of TiCl_2_ increases gas-phase collision frequency, promoting nucleation and subsequent growth of 2D MXene flakes. The as-synthesized lamellae self-organize into spherulites with diverse morphologies, forming a porous nanocrystal network consistent with the gas-to-solid process. The continuous lateral growth into larger flakes is observed as synthesis time increases, suggesting the feasibility of wafer-scale synthesis. This study provides a practical synthesis route for Ti_2_CCl_2_ MXenes, accelerating MXene research and applications in quantum technology, optics, and electronics, where 2D crystals with low defect density are required.

## Graphical abstract

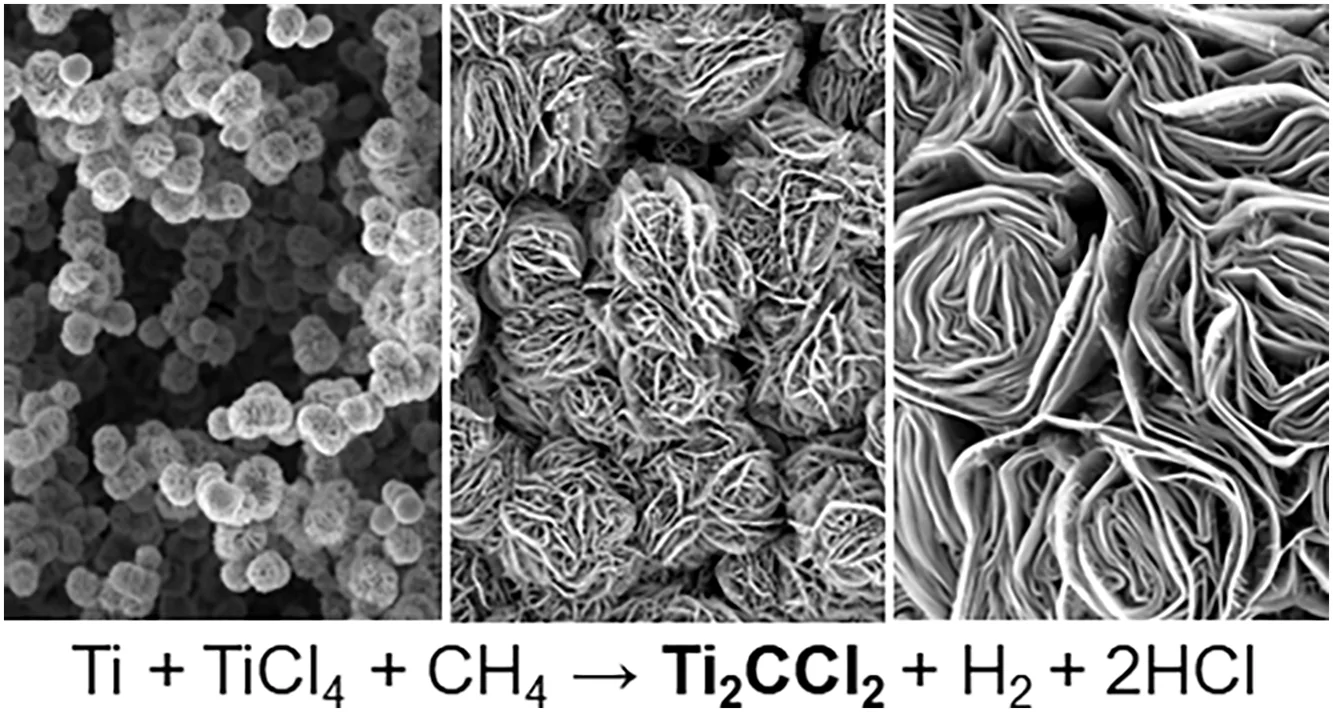

## Introduction

MXenes are the fastest-growing family of 2D materials. They have gained significant attention due to their structural diversity, compositional versatility, and unique property combinations.^1^ Their M-X frameworks and surface terminations (T_*x*_) provide powerful means of tuning electronic and chemical properties.^2–4^ Numerous synthesis approaches are based on selective etching of MAX phases and similar layered compounds in fluoride-containing acidic solutions,^5,6^ or Lewis-acid molten salts (LAMS).^7,8^ Recently, direct synthesis of M_2_XCl_2_ MXenes emerged as a potentially scalable and economical alternative,^9^ bypassing MAX-phase precursors, harsh etchants that produce defects, and excessive chemical waste. It allows the synthesis of nanosheets with uniform halogen terminations that can be further modified for systematic exploration of surface chemistry-property relationships and new applications. Despite these benefits, vapor-phase synthesis remains scarcely explored. Talapin et al. first established a chemical vapor deposition (CVD) process for MXenes,^9^ producing Ti_2_CCl_2_ carpets and microspheres on Ti foils by reacting TiCl_4_ and CH_4_ at 950 °C. This was followed by the fluidized bed furnace synthesis using TiCl_3_ and CH_4_ at 770 °C,^10^ and two-zone horizontal tube furnace synthesis of Ti_2_CCl_2_ and Ti_2_NCl_2_ by placing a porous Ti source in the hot zone at 1000 °C, supplying TiCl_4_ vapor upstream, and reacting it with CH_4_ or N_2_ at 770 °C.^11^ Utilization of organohalides (such as C_2_Cl_4_, C_2_Cl_6_, CH_2_Cl_2_, CBr_4_, and CH_2_Br_2_) has been considered as precursors for the direct synthesis of MXenes.^12^ The small number of published studies^9–12^ and lack of mechanistic clarity continue to constrain advances in vapor-phase MXene synthesis.

Thermodynamic studies have mainly focused on enthalpy-driven formation while underappreciating the impact of ternary intermediates and associated entropy gains. Ti can react with CH_4_ to form TiC at temperatures above 700 °C,^13^ as well as with TiCl_4_ to yield TiCl_3_ or TiCl_2_. The reaction between TiCl_3_ or TiCl_4_ and CH_4_ to yield TiC becomes thermodynamically favorable above 700 or 900 °C, respectively, when precursors are present at comparable concentrations.^14^ Disproportionation of TiCl_2_ to Ti and TiCl_4_ above 620 °C has been utilized for Ti thin-film deposition.^15^ At 1000 °C, Ti atoms supplied from TiCl_2_ react with C atoms from the decomposition of CH_4_ to form cubic TiC nanoparticles.^16^ Using the same precursors (Ti, TiCl_4_, CH_4_) under conditions nominally similar to the ones yielding Ti_2_CCl_2_, Ding et al. obtained nanoflower-shaped TiC, attributed to a relatively higher CH_4_ concentration.^17^ These observations emphasize the need to balance partial pressures of TiCl_*x*_ and CH_4_ and reaction temperature for Cl-terminated carbide MXene growth.

Here, we present a scalable vapor-phase synthesis of Ti_2_CCl_2_ MXene in a conventional tube furnace using Ti, TiCl_4_, and CH_4_. By confining the reaction volume and increasing the Ti surface area, we collected Ti_2_CCl_2_ spherulites composed of few-layer MXene lamellae on quartz substrates, confirming vapor-phase growth rather than surface-limited reactions. Mechanistic analysis reveals that exceeding a threshold TiCl_2_ concentration is essential for MXene nucleation and lateral growth, with extended reaction times producing larger flakes. The vapor-phase synthesized Ti_2_CCl_2_ MXene demonstrates sharp peaks in the Raman spectrum and high thermal stability.

## Results and Discussion

Placing Ti powder in a crucible and passing TiCl_4_ and methane (Figure 1a) resulted in the growth of Ti_2_CCl_2_ MXene on the top surface of Ti, forming a carpet and microspheres (Figure 1b), consistent with prior CVD growth on Ti foil.^9^ The underlying Ti particles predominantly underwent chemical etching. This limitation poses challenges for scaling up the synthesis. Chemical etching of Ti generates key TiCl_*x*_ intermediates (TiCl_3_ and TiCl_2_). Under laminar flow, TiCl_4_ is preferentially transported near the tube bottom, resulting in a reaction with Ti, but limiting reaction with CH_4_. Consequently, TiCl_*x*_ species react with CH_4_ primarily at the top Ti surface, localizing MXene growth. Notably, we formed MXene by using Ti sponge (from the Kroll process), which is nearly 4000 times less expensive than high-purity Ti foil (Figure S1 and Table S1).

**Figure 1:**
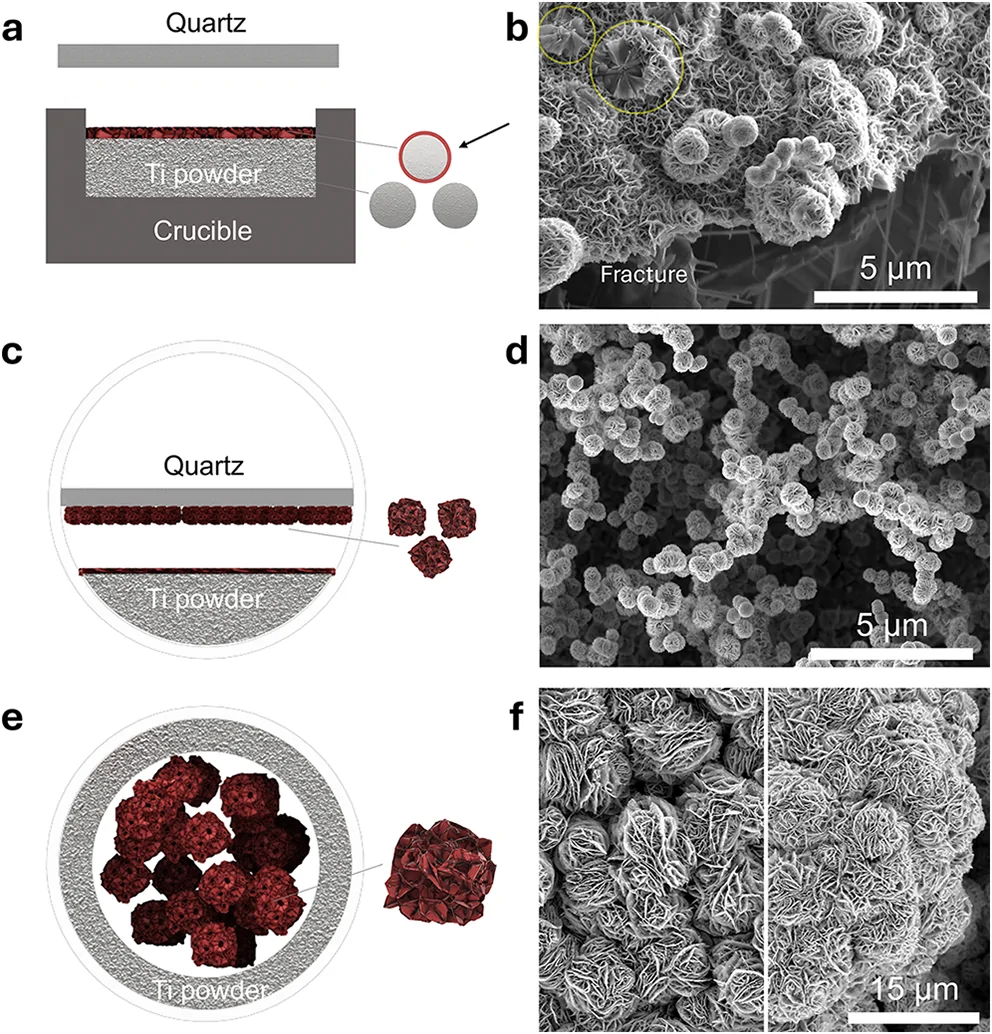
Schematic illustrations and scanning electron microscope (SEM) images of Ti_2_CCl_2_ MXene synthesized by using three different Ti loading methods. The cross-sectional view is shown in the left column. (a, b) Ti powder is loaded into a crucible. A thin layer of MXene was formed on the top surface of Ti, with a carpet and microspheres. Fractured microspheres are circled, showing the MXene flakes grown from the core with a lateral size equal to the spherulite radius. (c, d) Ti powder is loaded into a carrier quartz tube, and a quartz substrate is placed above it. MXene spherulites were deposited on the quartz substrate. (e, f) Ti powder is loaded on the inner surface of the carrier tube. After a 24-h reaction time, the volume inside the carrier tube was filled with MXene. The resulting MXene particles are intergrown spherulites, which merge into larger clusters.

To achieve vapor-phase growth independent of direct Ti contact, a quartz substrate was placed above the crucible. This approach led to the formation of a metallic Ti film with dispersed TiC particles on the substrate, rather than Ti_2_CCl_2_ MXene, indicating an insufficient TiCl_*x*_ concentration for MXene nucleation away from the Ti source. Confining the reaction volume with a quartz carrier tube (Figure 1c) increased precursor concentration and reduced diffusion length, resulting in the formation of black nanoparticle clusters on the quartz substrate facing the Ti powder, in addition to the MXene on the Ti bed. These subμm nanoparticles, hereafter referred to as *MXene spherulites*, assembled into a porous network typical of gas-to-solid reaction involving nucleation and growth (Figure 1d). Raman spectroscopy confirmed that the MXene spherulites formed on a quartz substrate were chemically identical, despite morphological differences. Formation of Ti_2_CCl_2_ on the quartz correlates with the balance between precursor generation and gas-phase diffusion, with volume confinement elevating TiCl_*x*_ partial pressure above the nucleation threshold. MXene spherulites grow on quartz within 1 h, with larger clusters forming after extended reaction times.

Distributing Ti powder along the inner wall of the carrier tube (Figure 1e) further enhanced vapor-phase MXene production, yielding bulk MXene through coalescence of adjacent spherulites (Figure 1f). The space within the carrier tube was filled with MXene after 24 h of reaction (Figure S2a). These results collectively demonstrate that controlling Ti loading and reaction volume is critical to achieving scalable vapor-phase growth and support earlier observations of vertical carpet and spherulites formed via gas-phase reaction of Ti subchlorides with CH_4_.^9^

Vapor-phase synthesis of Ti_2_CCl_2_ MXene involves reactions among four elements (Ti, Cl, C, and H), resulting in several possible binary intermediates with diverse stoichiometries. In this system, Ti undergoes chlorination and carburization, so maintaining a higher activity of chlorinated species than CH_4_ is essential, as lower Cl activity promotes cubic TiC formation over 2D Ti_2_CCl_2_. Maximizing TiCl_*x*_ production increases the activity of chlorinated species, thereby favoring MXene growth. While TiCl_3_ may form by thermal decomposition, reactions in the TiCl_4_–CH_4_–H_2_ systems yield no solids below 1000 °C and form TiC at higher temperatures.^10^ This suggests that TiCl_4_ decomposition, even in the presence of H_2_, cannot drive MXene formation without metallic Ti. We have performed experiments using pure Ar or 5% H_2_/Ar as chamber conditioning gas prior to deposition, and there was no significant difference in MXene synthesis. H_2_ can create a reducing ambient and therefore may react with supplied TiCl_4_ to yield TiCl_3_ and HCl. As detailed in Figure 2a, the main pathway is TiCl_2_ formation by Ti and TiCl_4_, followed by reaction with CH_4_ to yield Ti_2_CCl_2_ when TiCl_2_ exceeds a threshold concentration; otherwise, TiCl_2_ disproportionates back to Ti and TiCl_4_ as observed during the incubation stage. Elevated CH_4_ partial pressure or temperature favors TiC formation for all Ti species. Notably, Ti_2_CCl_2_ forms at a lower onset temperature than TiC, making high Cl activity critical for suppressing carburization.

**Figure 2:**
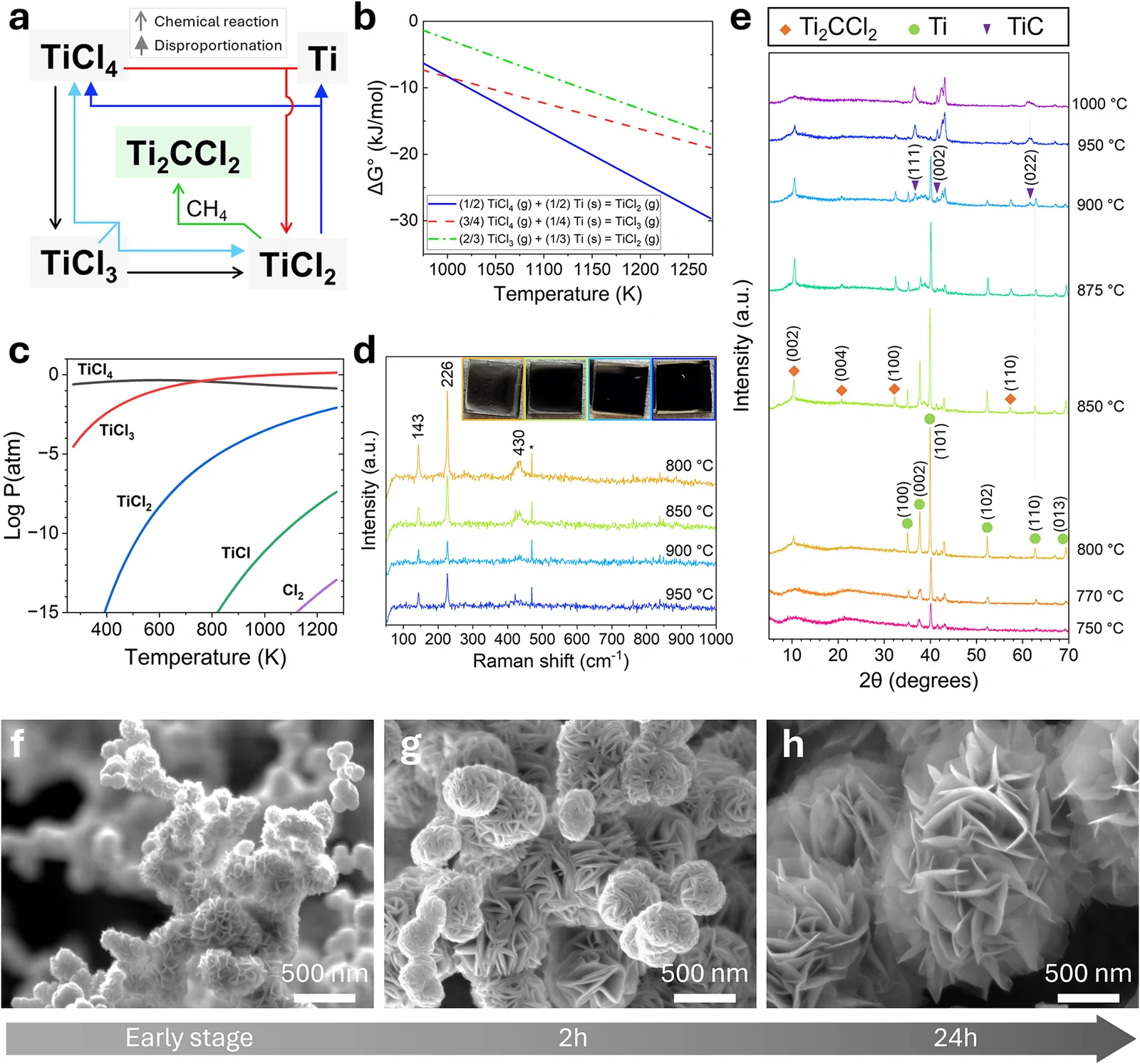
Thermodynamic analysis and characterization of Ti_2_CCl_2_ MXenes spherulites on quartz. (a) Schematic diagram of the reaction route leading to Ti_2_CCl_2_ MXene. We propose TiCl_2_ as the main MXene precursor from the primary reaction between Ti and TiCl_4_ (red). TiCl_2_ can form Ti_2_CCl_2_ MXene by reaction with CH_4_ (green) or disproportionate (blue), depending on TiCl_2_ concentration. All Ti compounds can form TiC when the C activity and/or temperature is high enough. (b) Calculation of Gibbs free energy changes for the reactions forming TiCl_2_ and TiCl_3_. TiCl_2_ is thermodynamically preferred over TiCl_3_ at temperatures above 1002 K. (c) Calculated saturated vapor pressures of TiCl_4_, TiCl_3_, and TiCl_2_. The calculation was performed for an equal molar ratio of Ti and Cl. (d) Raman spectra of samples produced at 800, 850, 900, and 950 °C. Asterisk (*) denotes hot pixel of the detector. Excitation: 633 nm. The inset images are photographs of MXene-deposited quartz plates measuring 1 × 1 cm^2^. (e) X-ray diffraction (XRD) patterns from reaction products obtained between 750 and 1000 °C. A metallic Ti layer was formed on the quartz substrate at 750–900 °C. Ti_2_CCl_2_ MXene appears at 800 °C and disappears at 1000 °C. Cubic TiC appears at 900 °C and above. (f–h) SEM images of Ti_2_CCl_2_ MXene spherulites grown at 950 °C. The early stage crystals are tiny particle aggregates (f), spherulites of approximately 300 nm in diameter are formed after 2 h reaction (g), and 2–3 μm-sized spherulites are formed after 24 h (h). Electron-beam-transparent MXene flakes in the spherulites are likely a few layers in thickness.

Thermodynamic calculations were performed to elucidate the reaction pathways between metallic Ti and TiCl_*x*_ species relevant to vapor-phase Ti_2_CCl_2_ formation. Figure 2b plots the temperature dependence of the Gibbs free energy changes for the following reactions with negative changes:

1 $$1/2{\;\mathrm{TiCl}}_{4}\left(g\right)+1/2\mathrm{Ti}\left(s\right)\to {\mathrm{TiCl}}_{2}\left(g\right)$$

2 $$3/4\;{\mathrm{TiCl}}_{4}\left(g\right)+1/4\mathrm{Ti}\left(s\right)\to {\mathrm{TiCl}}_{3}\left(g\right)$$

3 $$2/3\;{\mathrm{TiCl}}_{3}\left(g\right)+1/3\mathrm{Ti}\left(s\right)\to {\mathrm{TiCl}}_{2}\left(g\right)$$

Standard enthalpies and entropies were evaluated via temperature-dependent Shomate expressions with fitted coefficients in conjunction with the heat of formation. At *T* ≥ 1002 K, reaction ([Disp-formula eq1]) becomes more favorable than reaction ([Disp-formula eq2]). Additionally, TiCl_3_ (g) is further reduced to TiCl_2_ (g) by reaction ([Disp-formula eq3]). Notably, reaction ([Disp-formula eq1]) equals the fractional sum of reactions ([Disp-formula eq2]) and ([Disp-formula eq3]), reinforcing TiCl_2_ as the chemically privileged intermediate. By contrast, reactions producing TiCl (g) are strongly uphill in Gibbs free energy under the same conditions (Figure S3). Accordingly, the hypothetical pathway TiCl_4_ (g) + Ti (s) → TiCl_3_ (g) + TiCl (g) is not anticipated.

The calculated Ti–Cl binary phase diagram further supports the central role of TiCl_2_ (Figure S4), in agreement with the prior report.^15^ TiCl_2_ is more stable than TiCl_3_ at the growth temperature, and equilibrium speciation depends sensitively on the Ti:TiCl_4_ feed ratio. Yue et al. reported that TiCl_3_ becomes the dominant equilibrium species when the Ti:TiCl_4_ molar ratio is 1:3.^11^ However, TiCl_2_ dominates the equilibrium when the ratio shifts to 1:1 or 2:1.^18,19^ Consistent with this dependence, the density of MXene spherulites decreases in the most upstream regions of the reactor, where the accumulated TiCl_2_ concentration is relatively lower (Figure S5).

Figure 2c shows equilibrium partial pressures of TiCl_4_, TiCl_*x*_, and Cl_2_ calculated with TRAGMIN 5.2b software based on thermodynamic data.^20^ TiCl_4_ and TiCl_3_ are the predominant gaseous species, no matter the Ti:Cl ratio of 1:1 or 1:3, owing to the low saturated vapor pressure of TiCl_2_ and the thermodynamically disfavored TiCl and Cl_2_ formation. Under these conditions, the computed TiCl_*x*_ partial pressures approximate their saturated vapor pressure and are consistent with literature trends.^21–24^ Although the saturated vapor pressure of TiCl_2_ is orders of magnitude lower than that of TiCl_4_/TiCl_3_, reaction ([Disp-formula eq1]) continually generates TiCl_2_ vapor, enabling local supersaturation that drives its sublimation into the solid phase and subsequent reaction. Therefore, Ti_2_CCl_2_ MXene formation can proceed via TiCl_2_ supersaturation coupled with the intrinsic reactivity of TiCl_2_ toward CH_4_. The overall formation of Ti_2_CCl_2_ MXene follows the reaction ([Disp-formula eq4]).

4 $$2{\mathrm{TiCl}}_{2}\left(g\right)+{\mathrm{CH}}_{4}\left(g\right)\to {\mathrm{Ti}}_{2}{\mathrm{CCl}}_{2}\left(s\right)+2\mathrm{HCl}\left(g\right)+H_{2}\left(g\right)$$

TiCl_2_ reacts with methane to form Cl–Ti–Cl­(H_2_)–Ti–Cl structure with the release of HCl. H_2_ (g) in reaction ([Disp-formula eq4]) is a simplified byproduct accounting for the two excess hydrogen atoms that are hypothesized to terminate bonds to carbon atoms at the edge of MXene flake. These hydrogens are removed when the next “molecule” of Ti_2_CCl_2_ is added. A close-packed assembly of those molecules along the in-plane direction can form a 2D hexagonal structure with Cl-terminated surface, which is likely involved in the transition from tetrahedrally coordinated carbon (CH_4_) to octahedrally coordinated carbon (Ti_6_C octahedron) via band hybridization. The hypothetic growth mechanism requires further verification using computational methods. It is also interesting that the surface Ti–Cl bonds are maintained, without further reaction with CH_4_ toward TiC. The Ti_2_CCl_2_ solid contains Cl–Cl van der Waals interfaces, which can increase the system’s total energy but stabilizing Ti–Cl bonds on the surface of MXene flakes. Ti_2_CCl_2_ structure can be described as the thinnest hexagonal TiC crystal or as the close-packed (111) plane cut of cubic TiC with NaCl lattice and with maximum amount of Cl as termination. These terminations contribute to the entropy of mixing that can offset the additional interfacial energy. This MXene formation process can be attributed to the relative activity of carbon and chlorine at the deposition temperature (Figure S6).

Multiple experimental observations further implicate TiCl_2_ as the key gas-phase precursor: (i) Ti_2_CCl_2_ forms only near the Ti source, indicating a short transport length, consistent with the low saturated vapor pressure and thermal fragility of TiCl_2_. (ii) A metallic Ti layer is observed near the Ti source. TiCl_2_ vapor formed at the Ti surface diffuses a short distance, sublimates onto the quartz surface, and then decomposes back into solid Ti and TiCl_4_ during the initial incubation period. (iii) The molecular collisions favor the TiCl_2_ formation reaction between one TiCl_4_ molecule and one Ti atom, rather than that for TiCl_3_ involving three TiCl_4_ molecules colliding with one Ti atom. (iv) Heating TiCl_3_ solid is known to induce its disproportionation into TiCl_4_ and TiCl_2_,^23^ where the estimated vapor pressure of TiCl_2_ is an order of magnitude higher than that of TiCl_3_. Thus, formation of Ti_2_CCl_2_ MXene should be thermodynamically preferred over TiCl_2_ solid and TiC, especially at elevated Cl activity. The entropy of mixing can be maximized by forming a ternary compound and can dominate at higher temperatures. Such a reaction for forming Cl-terminated MXene requires a balanced activity of Cl and C in the reaction chamber.

Deposition experiments at 800–950 °C reveal high-quality MXene on quartz with characteristic Raman bands at 143, 226, and 430 cm^–1^ (Figure 2d). The same spectra were acquired from MXene formed on the Ti surface (Figure S1g). The two sharp Raman peaks at 143 and 226 cm^–1^ correspond to the E_g_ and A_g_ vibrational modes, respectively, dominated by Ti atoms. The relatively broad and weak peak at 430 cm^–1^ is attributed to a Cl termination**-**dominated mode. Only one peak from surface terminations was detected, suggesting either the overlap of E_g_ and A_g_ modes at nearby frequencies or the need for another laser source to resolve the other. No TiC_1–*x*_ or free carbon signal was detected between 1000 and 2000 cm^–1^ (Figure S7).

Figure 2e presents X-ray diffraction (XRD) patterns for Ti_2_CCl_2_ spherulites grown on a quartz substrate at 750–1000 °C for 2 h. At relatively lower temperatures (750–770 °C), the α-phase Ti was observed as a major product. Additionally, yellow-colored TiC_1*–x*_ formed near the edge of the substrate, and some black particles appeared at the boundary, which is likely Ti_2_CCl_2_ MXene (Figure S8). The low yield of MXene below 800 °C likely reflects limited concentration of precursor molecules and/or insufficient thermal activation. At 800 °C, the (002) peak of Ti_2_CCl_2_ MXene at 2θ∼10.5° is prominent. As the reaction temperature increases to 850 and 875 °C, the (002) peak becomes stronger, and the peaks corresponding to (004), (100), and (110) planes are also observed at 2θ∼20.8, 32.3, and 57.4°, respectively. Additional diffraction peaks are observed at 900 °C at 36.8, 41.4, and 61.8°, which correspond to the (111), (200), and (022) planes of cubic TiC, respectively. TiC may form in the core of a Ti_2_CCl_2_ spherulite where a local TiCl_2_-deficient environment is created. We confirmed the partial transformation of Ti_2_CCl_2_ MXene into TiC_1–*x*_, by stopping the TiCl_4_ supply at the growth temperature, which resulted in the evolution of a Raman response for TiC_1–*x*_ (Figure S6). Similar carburization can occur when Ti metal is completely consumed. At 950 °C, the Ti (101) and (110) peaks become weak and undetectable. It is noteworthy that the reaction yields Ti_2_CCl_2_ MXene at temperatures above 800 °C, overcoming the temperature limit of previously reported approaches at 770 °C.^10,11^ The higher reactivity of TiCl_*x*_ compounds relative to CH_4_ in our system enables high-yield MXene synthesis without the need to fluidize the source powder.

Above 900 °C, the *hcp* Ti peak diminishes, and the MXene spherulites network is often detached from the substrate. This observation indicates that the Ti film forms during the incubation period, when the concentrations of TiCl_2_ and CH_4_ are imbalanced, prior to the onset of steady MXene growth. At 1000 °C, most peaks corresponding to MXene and *hcp*-Ti disappeared, while TiC and *bcc*-Ti remained. Ti undergoes a phase transition between *hcp* and *bcc* structures at 882 °C. Since we utilized rapid cooling by opening the clamshell furnace, Ti remained in a metastable *bcc* phase. Considering Ti_2_CCl_2_ (002) peak intensity and the absence of TiC, 875 °C is the optimal synthesis temperature for a 2 h reaction. At lower reaction temperatures of 800 and 850 °C, the yield of MXene spherulites was lower. We observed hexagonal Ti grains on the quartz substrate, where reaction ([Disp-formula eq1]) occurs at the Ti powder surface, and the reverse reaction occurs at the quartz substrate, depositing a Ti thin film during the initial incubation stage. We also observed MXene formation at Ti grain boundaries (Figure S9), where the reaction ([Disp-formula eq4]) happens at a locally high-energy area.

SEM images for the initial, 2 h, and 24 h stages at 950 °C (Figure 2f–h) reveal multidirectional growth from initial nuclei. With prolonged time, spherulites open and reorient with flake edges aligning toward the incoming flux of TiCl_2_ and CH_4_. MXene spherulites show uniform size and morphology, similar to spherulitic architectures reported for MoS_2_ and MoSe_2_.^25,26^ Additional SEM images of MXene spherulites at low and high magnifications are provided in Figure S10. After 1 h, lamellae are densely packed, while a longer time produces pronounced flake elongation and separation. Increasing Ti loading in the carrier tube (Figure 1e) for 24 h fills the full cross-section with Ti_2_CCl_2_ and raises yield (Figure S2b). Morphologies span a continuum correlated with cluster size (Figure S11). The smaller MXene networks resolve into discrete hexagonal flakes with partial delamination, whereas larger aggregates, grown for longer durations, exhibit denser packing with increased flake bending and wrapping as space becomes constrained. The overall morphology resembles the microspheres in Figure 1b and the prior report,^9^ with lateral flake dimensions approaching the spherulite radius (See more examples in Figure S12). Possible mechanisms of MXene nucleation to a spherulite producing process, and the spherulite merging process are illustrated in Figure S13. The observed MXene carpet structure on Ti surface may have been originated from small-sized spherulites, which implies possible morphological modulations by controlling nucleation and growth rates.

Figure 3a reveals the distinctive swirl-like morphology that emerges from continuous vapor-phase MXene growth within a confined volume. Each domain originates as an individual spherulite, which expands and merges with adjacent spherulites, resulting in flakes reaching tens of micrometers in lateral dimensions. Figure 3b shows the XRD patterns of MXene powder synthesized at different temperatures over 24 h. The absence of metallic Ti peaks confirms the lack of a solid–gas incubation interface. At 950 °C, TiC peaks dominate with only a minor amount of Ti_2_CCl_2_. At 875 °C, the peak intensities of Ti_2_CCl_2_ and TiC become comparable, indicating a transition from MXene to TiC at longer reaction timeme. At 840–850 °C, MXene peaks are dominant, and TiC is much less prominent, with 850 °C identified as optimal for 24 h synthesis. Reaction at 840 °C did not suppress TiC formation but significantly reduced the synthesis yield. The small amount of TiC formation at a relatively lower temperature and over a longer time suggests that both thermodynamics and kinetics may be involved. Moreover, because prolonged reaction time results in enlarged MXene spherulites, the core region may have less local exposure to surrounding TiCl_2_ molecules. The relatively weak bonding energy of Ti–Cl and locally low Cl activity can cause partial loss of surface Cl, thereby making TiC formation preferred over Ti_2_CCl_2_. To completely avoid TiC formation, the synthesis scheme needs to change its morphology from spherulites to flat crystals or have the Cl-activity slightly higher. We expect more discoveries to follow for the vapor-phase synthesis of MXenes. The small amount of TiC is to be screened out during the delamination process, which equally requires further advances for a lower-cost and environmentally friendly approach.

**Figure 3:**
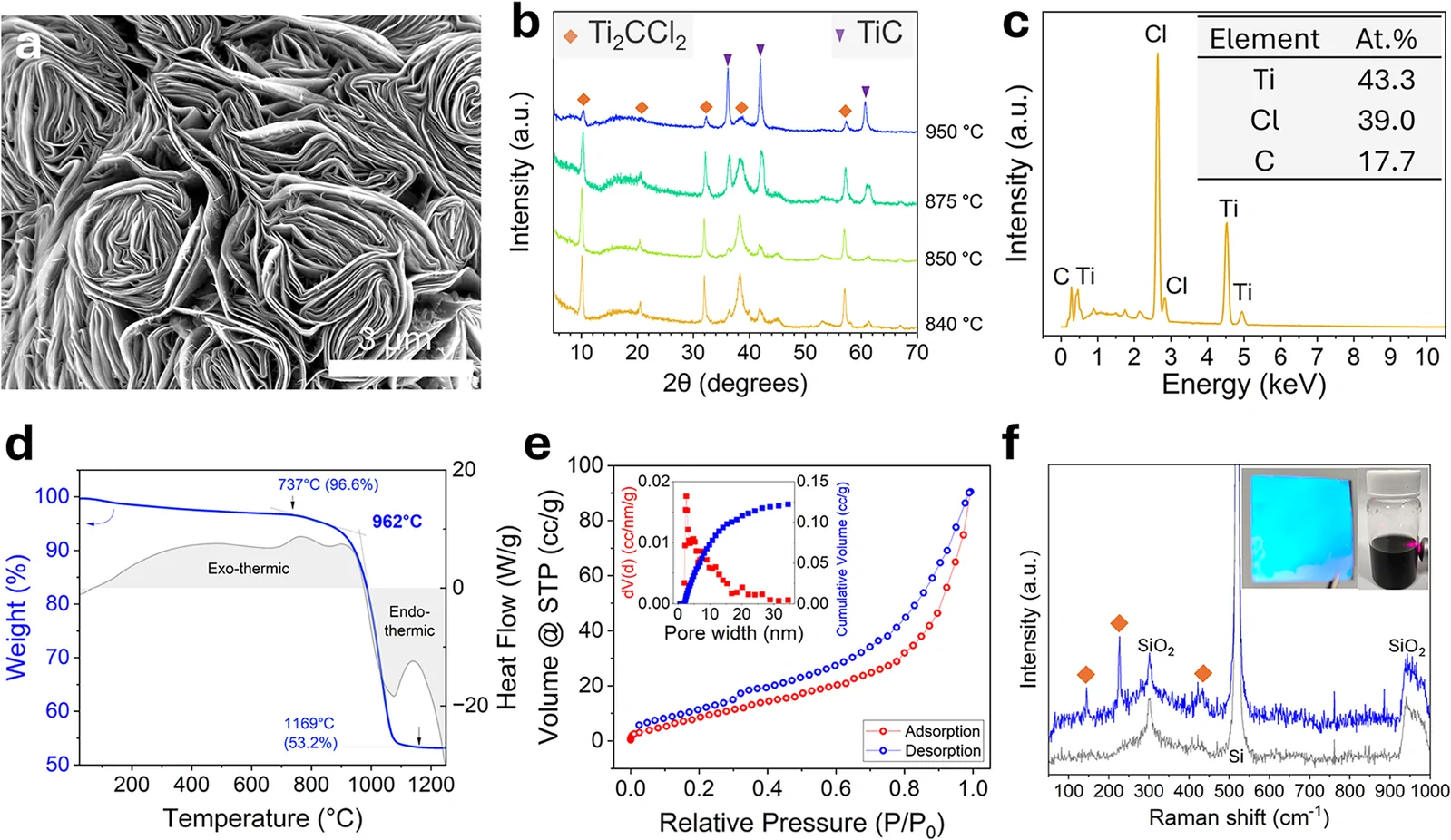
Characterization of Ti_2_CCl_2_ MXene powder synthesized via vapor-phase synthesis. (a) SEM image of bulk Ti_2_CCl_2_ resulting from the lateral growth of MXene flakes in a confined environment. (b) XRD patterns for reaction temperatures between 840 and 950 °C. Unlike the 2 h reaction (separated spherulites on quartz), the longer reaction above 850 °C resulted in the formation of TiC. This suggests a possible transformation of Ti_2_CCl_2_ into TiC under extended reaction time. (c) SEM-EDS spectra presenting the majority elements of Ti, Cl, and C. (d) TGA-DSC under Ar atmosphere with a heating rate of 10 °C min^–1^. (e) Nitrogen adsorption–desorption isotherm recorded at 77 K. The inset shows the pore size and volume distributions obtained by the QSDFT method. (f) Raman spectra of a delaminated Ti_2_CCl_2_ thin film on a SiO_2_/Si substrate prepared by the interfacial assembly. Inset images are photographs of MXene thin film and suspension in NMF.

SEM-EDS spectra in Figure 3c shows pronounced Ti and Cl signals, and the measured composition aligns closely with Ti_2_CCl_2_ stoichiometry. Figure 3d presents thermogravimetric analysis (TGA) of vapor-phase synthesized Ti_2_CCl_2_ powder under Ar, indicating thermal stability up to ∼900 °C; above this, mass loss occurs due to Cl release and decomposition into TiC. The observed 43.4% mass loss is consistent with the theoretical Cl content (39.7%). The thermal decomposition onset (962 °C) is comparable to reported LAMS Ti_3_C_2_Cl_2_ (999 °C),^4^ despite Ti_2_CCl_2_’s less stable M_2_C structure, and single-stage mass decrease at higher temperature suggests fewer defects versus the selectively etched MXene, as well as greater thermal stability than the O/OH/F terminated Ti_2_CT_*x*_ (∼700 °C)^27^ and Ti_3_C_2_T_*x*_ (∼850 °C).^28^ The N_2_ adsorption–desorption isotherm (Figure 3e) reveals mesopore-dominated porosity without a detectable micropore contribution. The multipoint Brunauer–Emmett–Teller (BET) surface area is 44.7 m^2^/g, and the pore volume from quenched solid density functional theory (QSDFT) is 0.12 cm^3^/g. Smaller-sized Ti_2_CCl_2_ spherulites exhibit a much higher specific surface area of 361 m^2^/g and pore volume of 0.71 cm^3^/g (Figure S14). We adopted an alternative Li^+^ intercalation method using organic solvent and LiCl salt,^29^ the powder was delaminated to yield a stable dispersion in n-methylformamide (NMF). The delamination yield was around 10% for both LiCl salt and *n*-butyllithium intercalation methods, and both successfully screened out the TiC. (Figure S15) Simple ultrasonication partially disentangled the small spherulites, revealing a porous network of few-layer MXene crystals, while the Li^+^ delaminated samples showed few- to monolayer flakes with tens to hundreds of micrometer sizes. The Raman spectra of the delaminated Ti_2_CCl_2_ thin film cast on a SiO_2_/Si substrate remain the same as the powder (Figure 3f), proving that the delamination does not destroy the MXene structure and surface groups.

Atomically resolved side-view high-resolution transmission electron microscope (HRTEM) images (Figure 4a,b) show few-layer lamellae exhibiting coherent epitaxial alignment across adjacent flakes, in contrast to MAX phase-derived MXenes, which often show alternating in-plane orientations due to A-element layers serving as twin boundaries.^30^ The absence of such boundaries in the vapor-phase synthesized MXene distinguishes bottom-up MXene crystals from the top-down counterparts produced via LAMS,^30^ or gas-phase etching of MAX phases.^31^ Basal-plane high-angle annular dark-field (HAADF)-scanning transmission electron microscope (STEM) images in Figure 4c,d resolve a honeycomb-like configuration of Ti and Cl atoms, described as Cl (A) – Ti (B) – C (Ti_6_C octahedron) – Ti (A) – Cl (B) stacking, where A and B denote close-packed planes. At the Cl–Cl van der Waals interface, the adjacent Cl layer occupies the hollow sites of the surface Cl layer, forming a *fcc*-like alternating sequence that matches the contrast observed in Figure 4b,d. The corresponding fast Fourier transform (FFT) image in the inset confirms the hexagonal atomic symmetry of monolayer Ti_2_CCl_2_. Notably, our experimental atomic observation and Raman data agree well with the theoretically simulated atomic structure and phonon dispersion of Ti_2_CCl_2_.^32^

**Figure 4:**
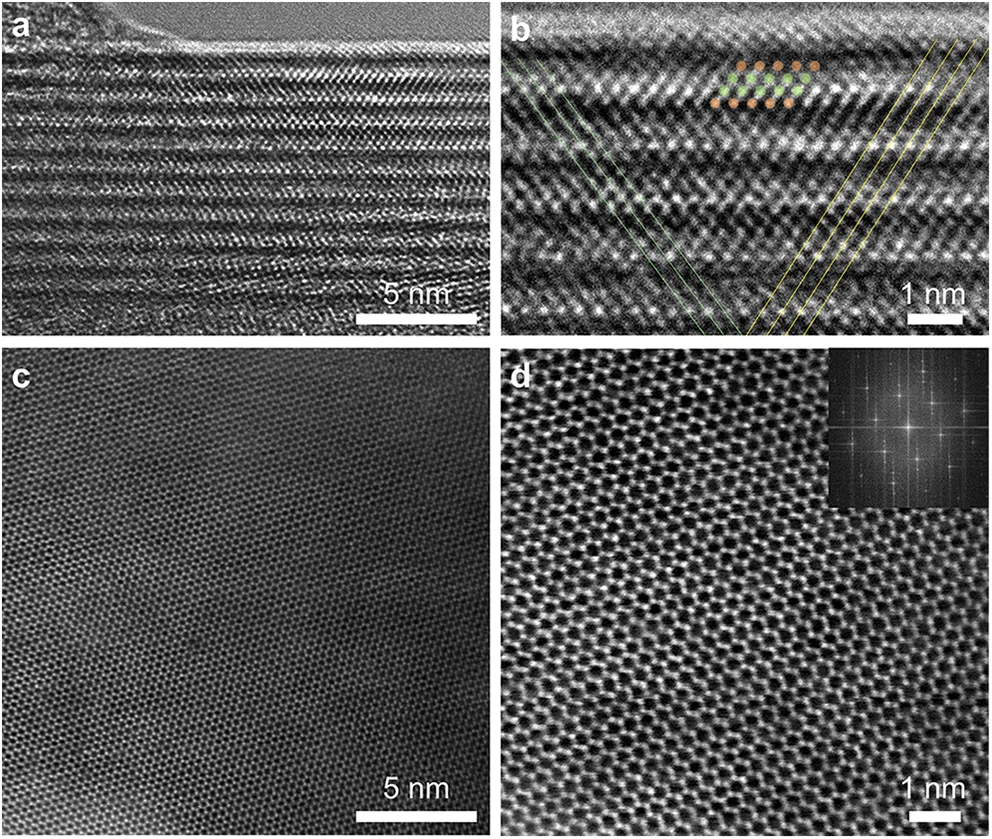
TEM analysis of vapor-phase synthesized Ti_2_CCl_2_ MXene at low and high magnification. (a, b) Atomically resolved cross-sectional HRTEM images. There is a registry between MXene flakes in a stack. The yellow and green lines are shown to guide the eye. Green and orange circles in (b) denote Ti and Cl atoms, respectively. (c, d) Atomically resolved HAADF-STEM images with a zone axis of [0001] for a honeycomb-like basal plane configuration. The inset is a fast Fourier transform (FFT) image showing the hexagonal symmetry.

## Conclusions

A scalable vapor-phase synthesis route to Ti_2_CCl_2_ is established by precisely modulating precursor activities in a tube furnace, yielding few-layer MXene flakes arranged into spherulites formed via gas-phase nucleation and growth. The resulting open architecture exhibits a high surface area, predominantly characterized by mesoporosity. Systematic studies reveal that the partial pressure of TiCl_2_ is the critical parameter controlling nucleation density, growth rate, and product morphology. By substituting high-purity Ti foil with low-cost Ti powder or sponge and confining the reaction volume, the intermediate molecular concentration has been enhanced, thus promoting gas-to-solid MXene formation. Reaction temperature and duration offer additional tuning options for MXene flakes and spherulite sizes, thereby controlling the accessible surface area, which is important for gas adsorption, desalination, catalysis, and energy storage. The sensitivity of nucleation to TiCl_2_ activity provides a pathway to single-flake MXene growth by lowering the steady-state TiCl_2_ concentration to suppress secondary nucleation while sustaining lateral growth. These insights lay a foundation for achieving in-plane growth of large-area, low-defect Ti_2_CCl_2_ MXene, marking an important advance toward wafer-scale monolayer synthesis for emerging electronic and quantum devices. The vapor-phase route eliminates the need for MAX phase precursors, extensive postgrowth washing or chemical treatment, unless a chemical modification or delamination is required, thus minimizing chemical waste generation and producing MXene of the highest crystal quality compared to other synthesis methods.

## Experimental Section

### Chemicals and Materials

Ti foil (≥99.99%, thickness 0.127 mm, Millipore Sigma), Ti powder (−325 mesh, 99.5%, Thermo Scientific Chemicals), Ti sponge (Pyro Chemical Source LCC), TiCl_4_ (99.9%, Millipore Sigma), Acetone (99.5%, Millipore Sigma) were used as purchased without further purification.

Ar gas (ultrahigh purity grade, Airgas), Ar/CH_4_ gas (5% CH_4_, ultrahigh purity grade, Airgas) were supplied by mass flow controller units from Alicat Scientific. Carrier quartz tube (10 mm I.D. × 12 mm O.D. × 4 cm length) was purchased from Technical Glass Products, Inc. Quartz substrate (JGS2, 4-in wafer, 500 μm thick) was purchased from WaferPro and diced into 10 mm × 10 mm.

#### Vapor-Phase Synthesis of Ti_2_CCl_2_

1.8 g of titanium powder or sponge was dispersed into 10 mL of acetone. A major part of the sedimented Ti powder was transferred into two quartz carrier tubes (10 mm I.D., 4 cm length) using a plastic transfer pipette. Two 10 × 10 mm^2^ quartz substrates (JGS2) were inserted into each carrier tube. The samples (the carrier quartz tubes with Ti powder and optional quartz substrates) were loaded into the heating zone of a horizontal tube furnace (1-in diameter work tube). The chamber was filled with Ar before heating by alternating purging and vacuum cycles three times and by the continued flow of 200 sccm for 20 min. The furnace was heated to 800 °C (ramping at 30 °C/min, hold for 2 min), then heated to the target temperature at 10 °C/min, and the temperature was maintained for the desired reaction time of 2–24 h. Once the target temperature was reached, the Ar supply was bypassed via a TiCl_4_ bubbler (1.7% by saturated vapor pressure at room temperature), and the desired Ar/CH_4_ (5% CH_4_) flow was introduced in parallel. After the deposition, the Ar flow was adjusted to the original path into the chamber directly (without TiCl_4_ supply), and the Ar/CH_4_ flow was stopped. The splitable furnace lid was opened for rapid cooling. Ar flow was applied continuously overnight. The sample was collected without further washing or chemical treatment and stored in a vial on the laboratory benchtop. The delamination process was reported in our earlier study using organic solvents and LiCl salt.^29^

### Material Characterization

The samples were characterized by X-ray diffraction (XRD), Raman spectroscopy, scanning electron microscopy (SEM), energy-dispersive X-ray spectroscopy (EDS), transmission electron microscopy (TEM), and other methods.

#### XRD

The diffraction patterns were obtained on a MiniFlex benchtop diffractometer (Rigaku), with a Cu Kα X-ray source (1.5406 Å) operating at 40 kV and 15 mA. The samples on the quartz substrate were measured with the substrate in place, which was also applied to the sample holder to match the height.

#### Raman Spectroscopy

Raman spectra were acquired by a LabRAM HR Evolution confocal microscope (Horiba). A Si wafer was used for autocalibration before each measurement session. The sample was excited by a 633 nm laser source with a × 100 objective lens and a 600 lines/mm grating. The measurements were performed with a 3.2% filter for 60 s. The cosmic rays were eliminated by recording each spectrum twice. The sample was unaffected by laser exposure.

#### SEM-EDS

SEM and EDS analyses were performed using Apreo S2 SEM (Thermo Fisher) with an acceleration voltage of 5 kV (SEM) and 10 kV (EDS) in OptiPlan mode. The working distance was 10 mm. Trace amount of Si, Au, and Al was detected for a sum of less than 1% (from Au-coated SiO_2_/Si substrate and Al-stub), and that contribution was excluded.

#### TEM

The sample for TEM analysis was prepared by filtering a 10 μL mixture of MXene spherulites and ethanol multiple times through lacey carbon on copper grids. The preliminary high-resolution transmission electron microscopy (HRTEM) analysis was performed on a JEOL F200 operating at 200 kV. The HRTEM images were acquired with a Gatan Oneview camera at a resolution of 4096 × 4096. High-angle annular dark-field scanning transmission electron microscopy (HAADF-STEM) was conducted on an aberration-corrected JEOL NEOARM operating at 200 kV. The HAADF-STEM images were acquired using 160 pA probe current, a convergence semiangle of 25 mrad, and an exposure time of 12 μs per pixel. The HAADF detector set at 4 cm camera length had an inner collection semiangle of 35.8 mrad and an outer collection semiangle of 127 mrad. The Richardson-Lucy filter was applied to the HAADF-STEM images.

#### Other Characterizations

Thermogravimetric analysis (TGA) coupled with differential scanning calorimetry (DSC) was performed using a Discovery SDT650 under Ar at a heating rate of 10 °C min^–1^. Brunauer–Emmett–Teller (BET) analysis was conducted by the Quadrachrome Autosorb iQ using nitrogen gas at liquid nitrogen temperature. The sample was degassed at 200 °C under vacuum before the measurement.

#### Thermodynamic Calculations

The Gibbs free energy minimization for reactions between Ti (s), TiCl (g), TiCl_2_ (g), TiCl_3_ (g), and TiCl_4_ (g) was performed by considering both enthalpy and entropy of known chemicals, as well as the heat of formation.

$$\Delta G^{{}^{\circ}}=\Delta H^{{}^{\circ}}-T\Delta S$$

$$\Delta H^{{}^{\circ}}=\Sigma H^{{}^{\circ}}\;\left(p\mathrm{roducts}\right)-\Sigma H^{{}^{\circ}}\;\left(r\mathrm{eactants}\right)$$

$$H_{T}^{{}^{\circ}}=\left[H^{{}^{\circ}}-H_{298.15}^{{}^{\circ}}\right]+\Delta_{f}H_{gas}^{{}^{\circ}}\;\left(h\mathrm{eat}\;\mathrm{of}\;\mathrm{formation}\right)$$

$$\Delta S^{{}^{\circ}}=\Sigma S^{{}^{\circ}}\;\left(p\mathrm{roducts}\right)-\Sigma S^{{}^{\circ}}\;\left(r\mathrm{eactants}\right)$$

Gas phase thermochemistry data, including [*H*° – *H*°_298_], *S*°, and Δ_f_ *H*°_gas_ data, were taken from NIST Chemistry WebBook,^33^ by coefficients with the Shomate equation as a function of temperature, unless specifically mentioned otherwise. The heat of formation for TiCl_2_ (g) was obtained from the heat of formation of TiCl_2_ (s)^34^ and the enthalpy of sublimation of TiCl_2_ (s).^35^ The heat of formation for Ti (s) is zero.

The vapor pressures of TiCl_*x*_ were calculated by TRAGMIN 5.2b software.^20^ The simulation conditions were iterated within a reasonably small volume until similar results were obtained at Ti: Cl molar ratios of 1:1 to 1:3, to ensure that the results represent the saturated vapor pressures of TiCl_*x*_.

## Supplementary Material



## Notes

SEM images and Raman spectra of Ti powder and Ti sponge after reaction, unit price comparison for Ti precursors, photographs of carrier tubes with different Ti powder loading methods (before and after 24 h reactions), calculated Gibbs free energy changes for TiCl_*x*_. Ti–Cl binary phase diagram calculated by ThermoCalc software. Photograph of quartz samples with Ti_2_CCl_2_ MXene coating. Schematic illustration of the possible reaction route between TiCl_2_ and CH_4_. Raman spectra of Ti_2_CCl_2_ with and without intended carburization for partial phase transformation into TiC_1–*x*_. Photographs and SEM images of quartz samples at low temperatures (750 and 770 °C) with clear boundaries between Ti, Ti_2_CCl_2_, and TiC_1–*x*_. SEM images of the quartz sample after 2 h of reaction at 800 and 850 °C. SEM images of MXene spherulites deposited at 950 °C for 1, 2, 4, and 24 h. SEM images of bulky Ti_2_CCl_2_ MXene grown at 840 and 850 °C for 24 h. SEM images of fractured MXene spherulites showing flake growth direction. Schematic illustration of MXene spherulites formation and merging process. BET analysis of Ti_2_CCl_2_ MXene spherulites. TEM images of Ti_2_CCl_2_ MXenes with ultrasonication and delamination with LiCl and *n*-butyllithium (PDF)
