# Supplementary material for: Vapor-Phase Synthesis of Ti2CCl2 MXene
Source: J Am Chem Soc. 2026 Aug 17;148(40):43191–9. doi: 10.1021/jacs.6c10774 (PMC13674082; doi:10.1021/jacs.6c10774)
Supplement: Supplementary file 1 [file ja6c10774_si_001.pdf]

Supporting information for

Vapor-Phase Synthesis of Ti_2CCl_2 MXene

Hyunho Kim[1,4], Jongyoun Kim[1], Teng Zhang[1], Swarnendu Das[2], Yasunori Hioki[1,3], Eric A. Stach[2], Yury Gogotsi[1*]

[1] A. J. Drexel Nanomaterials Institute and Department of Materials Science and Engineering, Drexel University, Philadelphia, Pennsylvania 19104, United States

[2] Department of Materials Science and Engineering, University of Pennsylvania, Philadelphia, Pennsylvania 19104, United States

[3] Murata Manufacturing Co., Ltd., 1-10-1 Higashikotari, Nagaokakyo-shi, Kyoto, 617–8555 Japan

[4] School of Advanced Materials Science and Engineering, Sungkyunkwan University, Seobu-Ro 2066, Jangan-Gu, Suwon-Si, Gyeonggi-Do, 16419, Republic of Korea

* Corresponding author. Email: gogotsi@drexel.edu

Figure S1. SEM images and Raman spectra of Ti powder and Ti sponge after reaction.

Table S1. Unit price comparison for Ti precursors.

Figure S2. Photographs of carrier tubes with different Ti powder loading method (before and after 24 h reactions).

Figure S3. Calculated Gibbs free energy changes for $TiCl_x$.

Figure S4. Ti-Cl binary phase diagram calculated by ThermoCalc software.

Figure S5. Photograph of quartz samples with Ti_2CCl_2 MXene coating.

Figure S6. Schematic illustration of possible reaction route between $TiCl_2$ and CH_4.

Figure S7. Raman spectra of Ti_2CCl_2 with and without intended carburization for partial phase transformation into TiC_{1-x}.

Figure S8. Photographs and SEM image of quartz samples at low temperatures (750 and 770°C) with clear boundaries between Ti, Ti_2CCl_2, and TiC_{1-x}.

Figure S9. SEM images of quartz samples after 2 h reaction at 800°C and 850°C.

Figure S10. SEM images of MXene spherulites deposited at 950°C for 1, 2, 4, and 24 h.

Figure S11. SEM images of bulky Ti_2CCl_2 MXene grown at 840 and 850°C for 24 h.

Figure S12. SEM images of fractured MXene spherulites showing flake growth direction.

Figure S13. Schematic illustration of MXene spherulites formation and merging process.

Figure S14. BET analysis of Ti_2CCl_2 MXene spherulites.

Figure S15. TEM images of Ti_2CCl_2 MXenes with ultrasonication and delamination with LiCl and n-butyllithium.

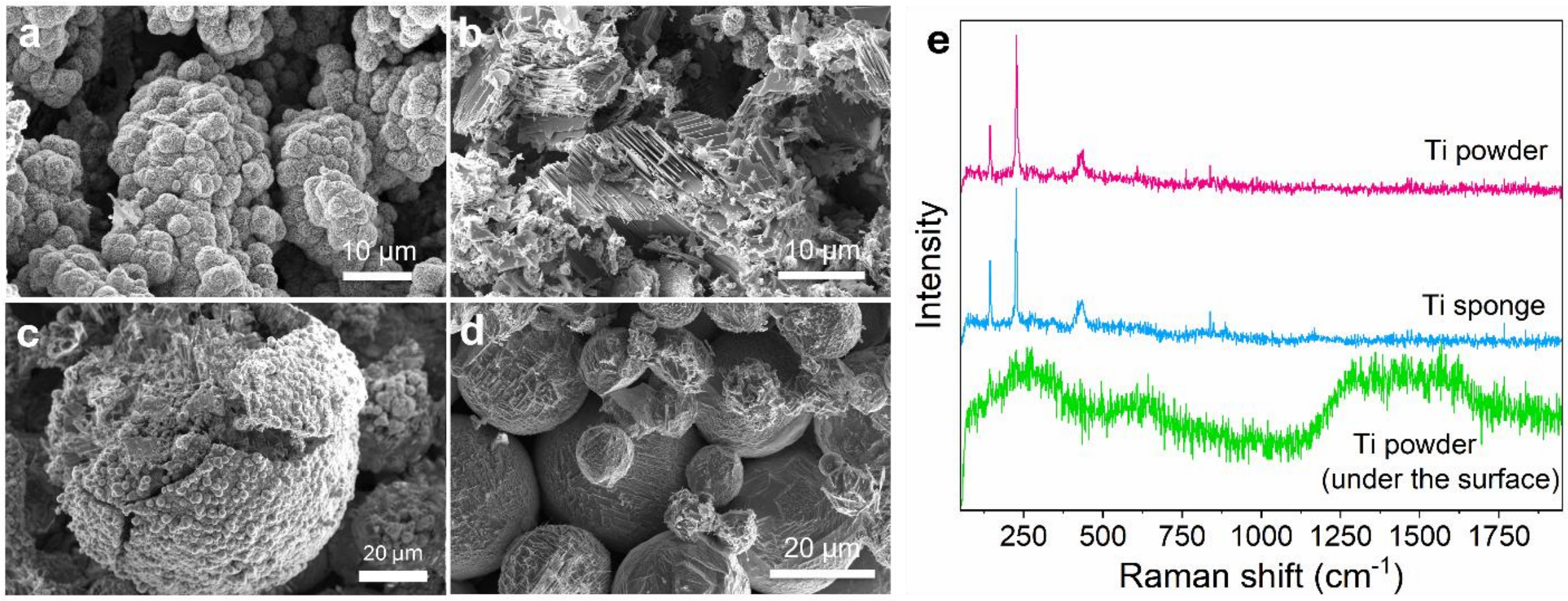

Figure S1. SEM images and Raman spectra of the products synthesized using Ti powder and Ti sponge loaded in a crucible. **a)** 99.5% purity Ti powder after reaction, coated by MXene. **b)** The same batch of titanium powder underneath the surface. Certain facets of the titanium grains are exposed because of chemical etching through preferred orientation. **c)** Ti sponge coated by MXene after reaction. This is a lower-magnification version of the image shown in Fig.1b near fracture. **d)** The same batch of Ti sponge underneath the surface. The rounded particles expose certain facets with steps because of chemical etching. **e)** Raman spectra collected from the top surface of Ti powder and Ti sponge after 2 h reactions that match Ti_2CCl_2 MXene. The remaining Ti powder underneath the surface did not show such Raman response. Data shown here represent the method described in Fig. 1a.

Table S1. Unit price comparison for Ti precursors.

	Provider	Purity (%)	Price (USD)	Unit (g)	Unit price ($/g)
**Ti Foil**	Aldrich	≥ 99.99	288	1.5	192
**Ti Powder**	Thermo Fisher	99.5	249.95	500	0.50
**Ti Sponge**	Pyro Chemical Source LLC	unknown	20	453	0.044

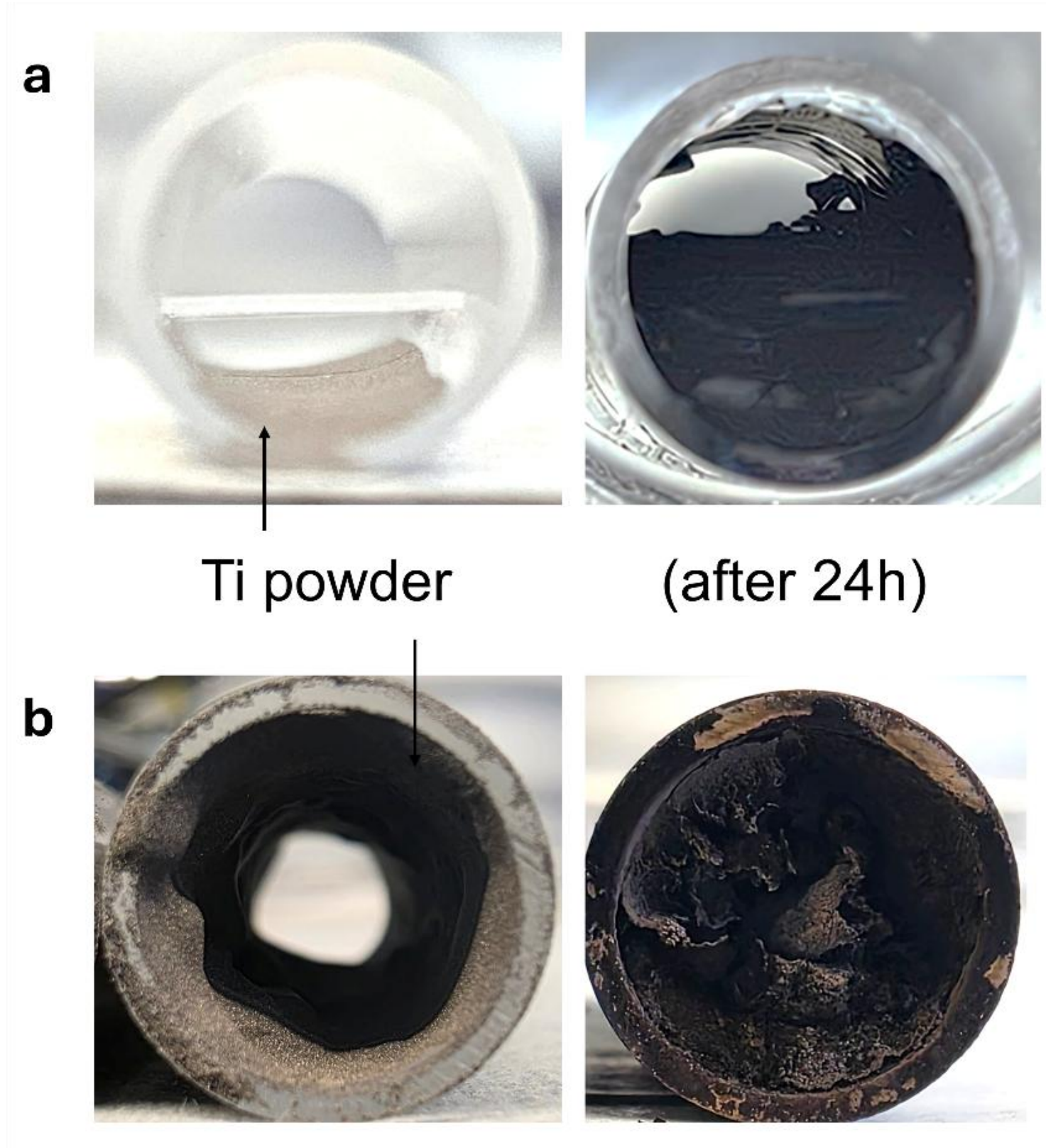

Figure S2. Digital photographs of samples before (images on the left) and after (images on the right) 24 hours of reaction. **(a)** Ti powder and quartz substrate loaded in a quartz carrier tube. **(b)** Ti powder covering the entire inner surface of the quartz carrier tube. (a) and (b) represent the methods described in Fig. 1c and Fig. 1e, respectively.

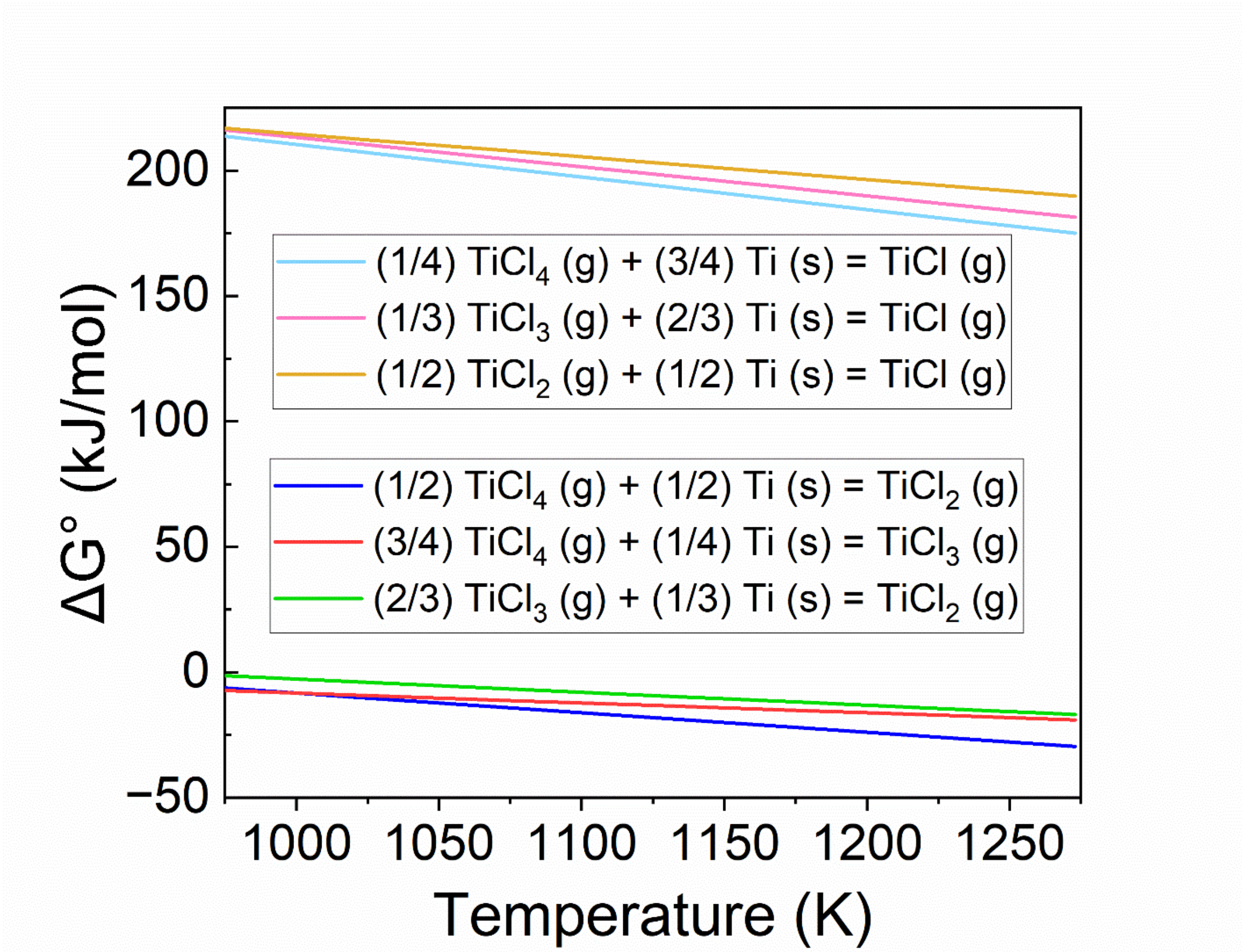

Figure S3. Calculated Gibbs free energy changes for 6 possible reactions. The formation of TiCl (g) is not thermodynamically favorable, unless coupled with other reactions consuming TiCl (g). The result suggests that $TiCl_2$ (g) can be the primary byproduct from the reaction between Ti (s) and $TiCl_4$ (g) at above 1002 K.

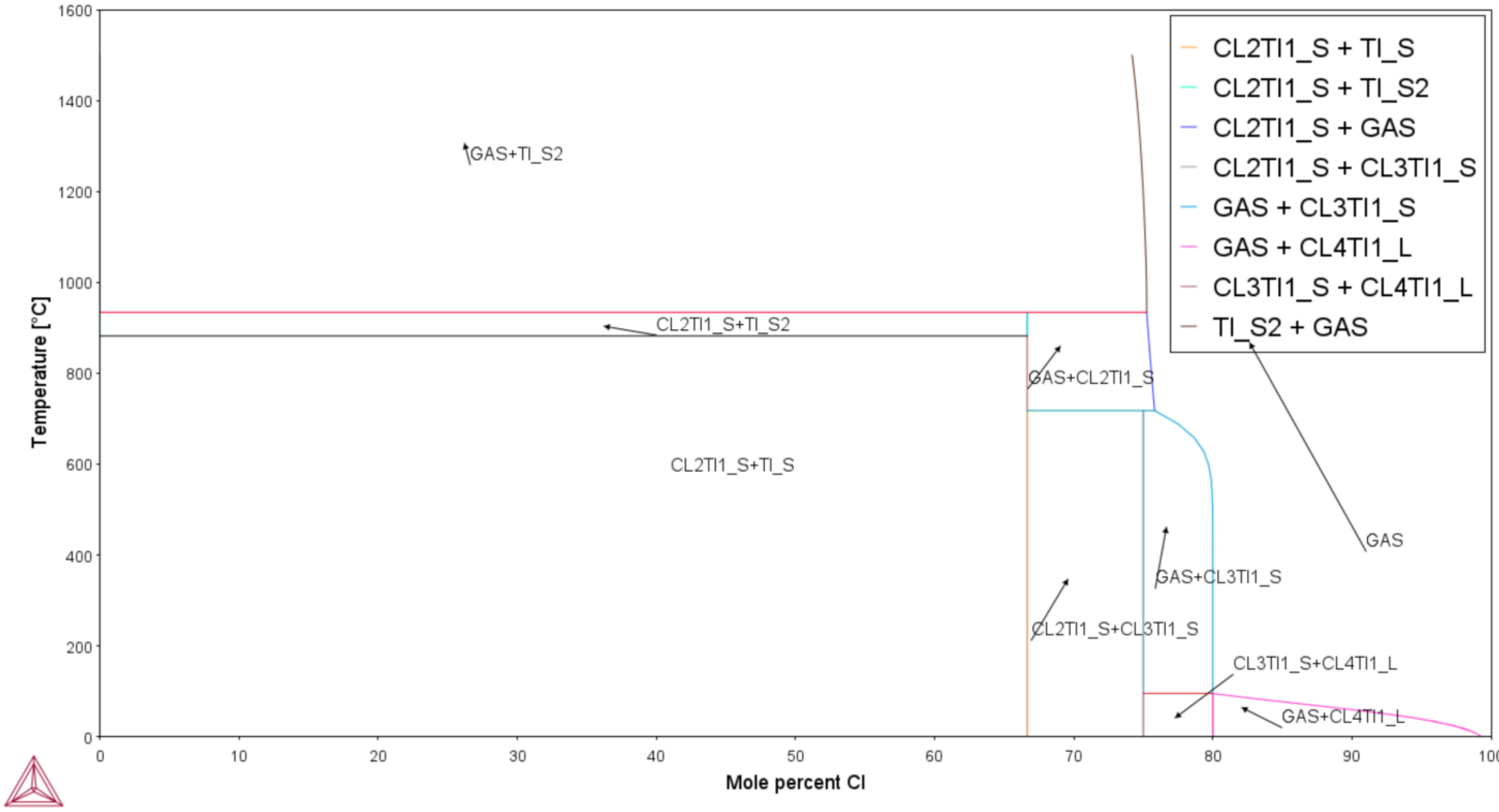

Figure S4. Ti-Cl phase diagram obtained by ThermoCalc. At temperatures above around 710°C, $TiCl_3$ (vertical line at 75 mol% Cl) is no longer the most stable product. $TiCl_2$ (vertical line at 66 mol% Cl) becomes thermodynamically preferred. This diagram also supports the possible decomposition of $TiCl_2$ into Ti and $TiCl_4$ molecules, depending on the local activity of titanium chloride vapor in the reaction tube.

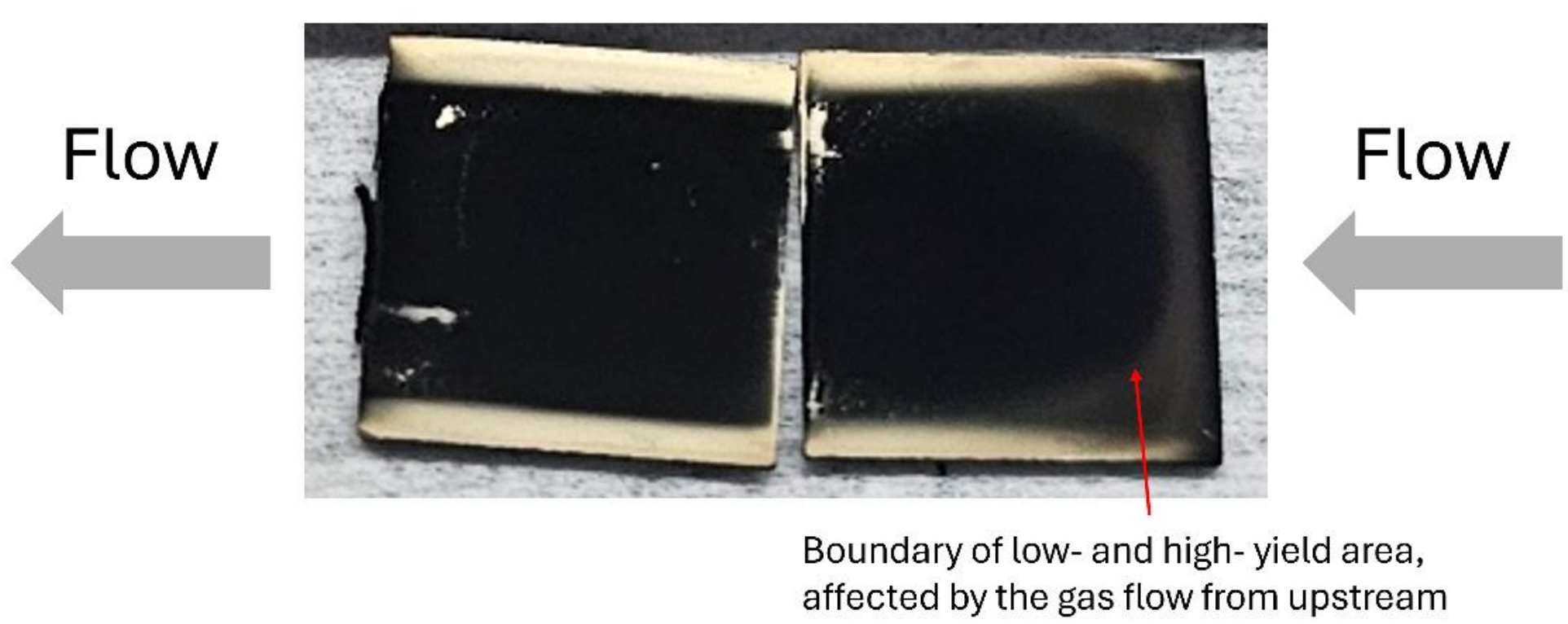

Figure S5. Digital photograph of Ti_2CCl_2 MXene spherulites coating deposited on 1×1 cm^2 quartz substrates. The right-hand side is the upstream where the mixed gas first meets the Ti source (flow from the right to the left). The MXene formation area is well visible with clear boundaries. The yellow-golden colored edge areas are where the quartz substrate contacts the carrier tube. Those are likely TiC_{1-x}, a carburized titanium film.

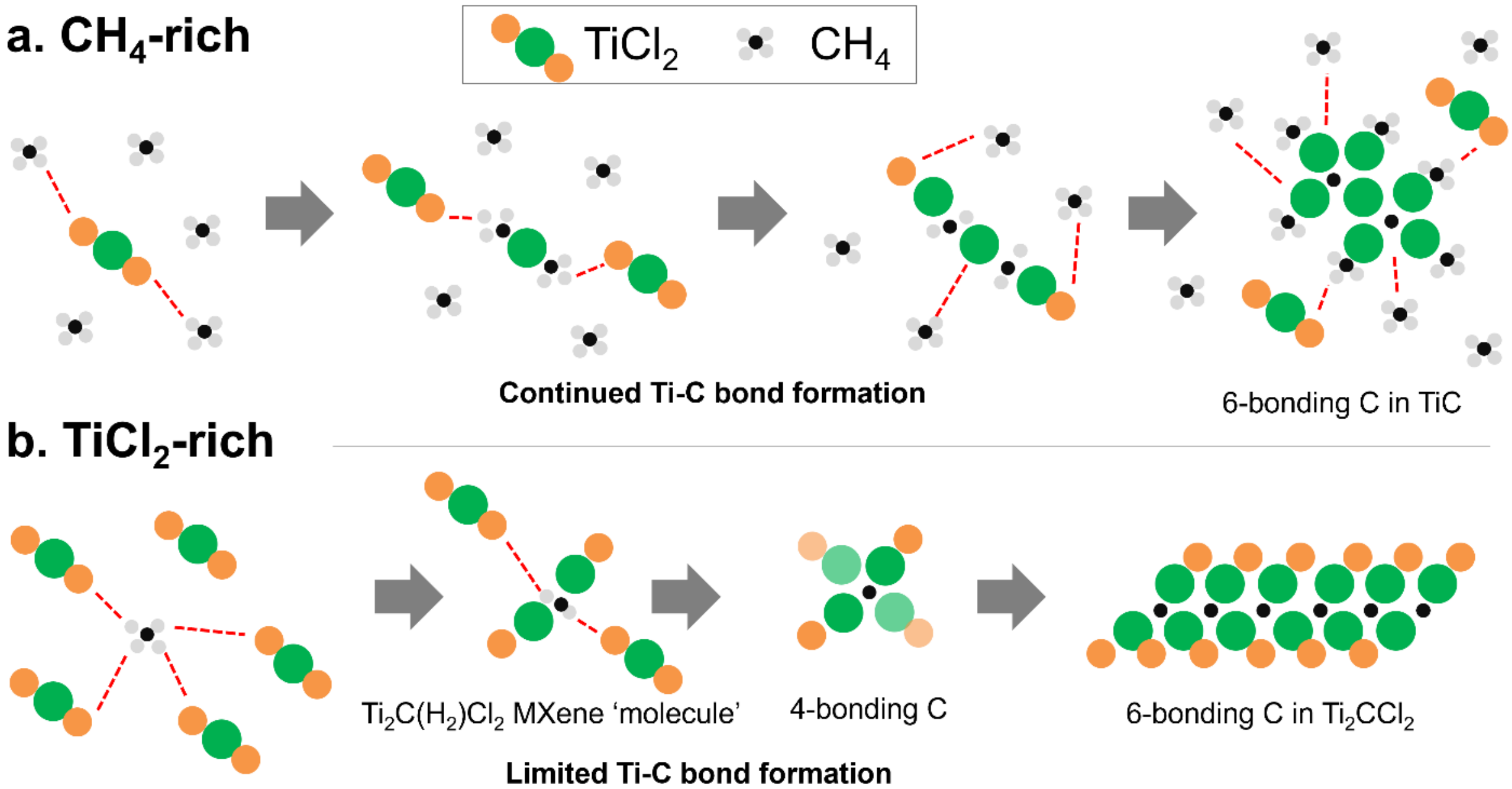

Figure S6. Schematic illustration of possible reaction route between $TiCl_2$ and CH_4. **(a)** Under CH_4-rich ambient. The relatively unstable $TiCl_2$ molecule react with abundant CH_4, resulting in cubic TiC. **(b)** Under $TiCl_2$-rich ambient. The methane molecule connects $TiCl_2$ molecules around by forming limited Ti-C bonds. Such route can preserve the surface Ti-Cl bonds.

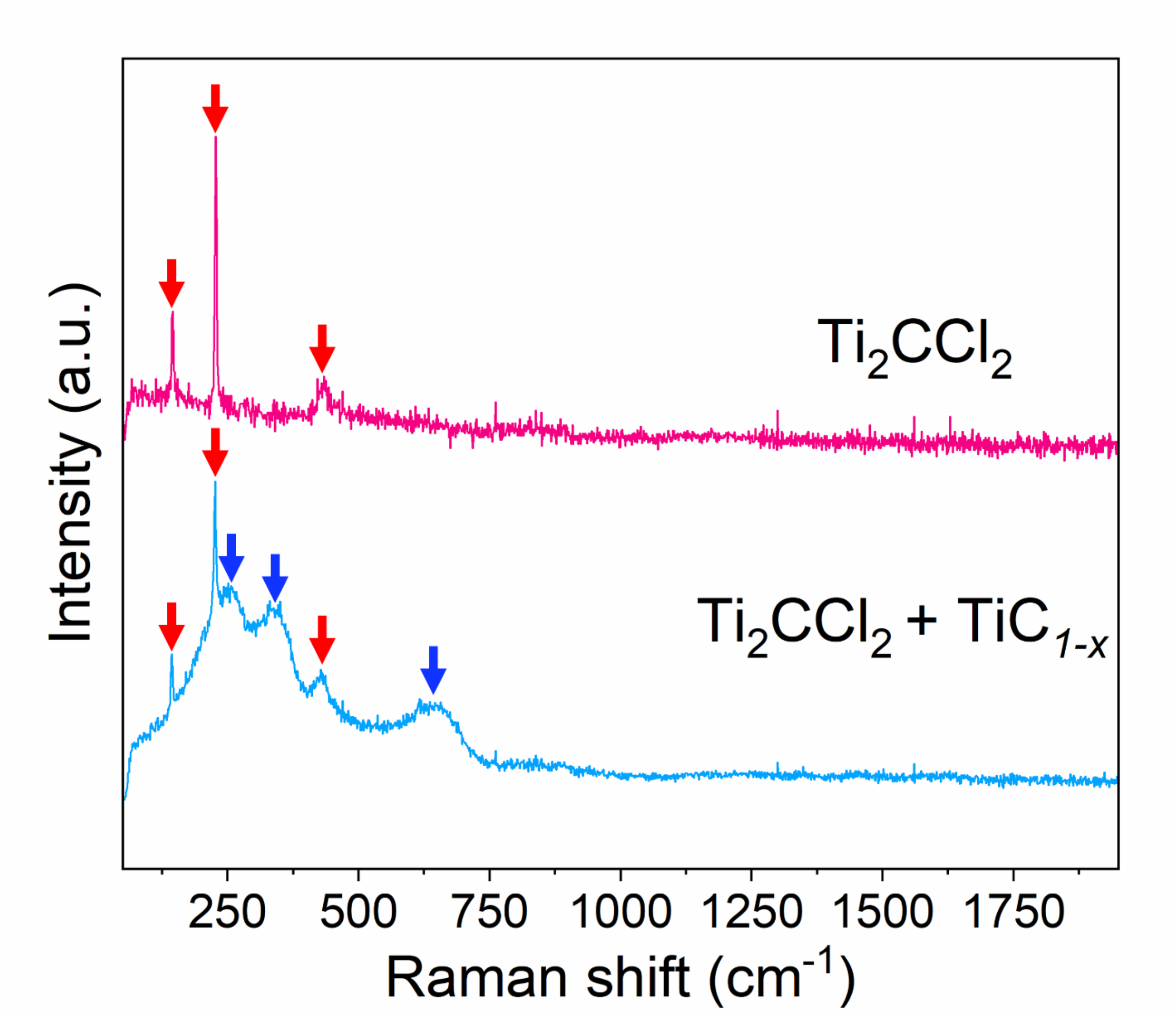

Figure S7. Raman spectra of a "successful" synthesis that led to pure Ti_2CCl_2 and an "unsuccessful" one that produced Ti_2CCl_2 with a significant amount of TiC_{1-x}. Ti_2CCl_2 has three Raman peaks (red arrows). In the case of "unsuccessful" samples, TiC_{1-x} response (blue arrows) appears with two bands at lower (200-400 cm^{-1}) and higher (500-700 cm^{-1}) Raman shifts overlapped with MXene response. The "unsuccessful" synthesis resulted from stopping the $TiCl_4$ supply at growth temperature, which led to partial transformation of Ti_2CCl_2 into TiC_{1-x}. No free carbon was observed.

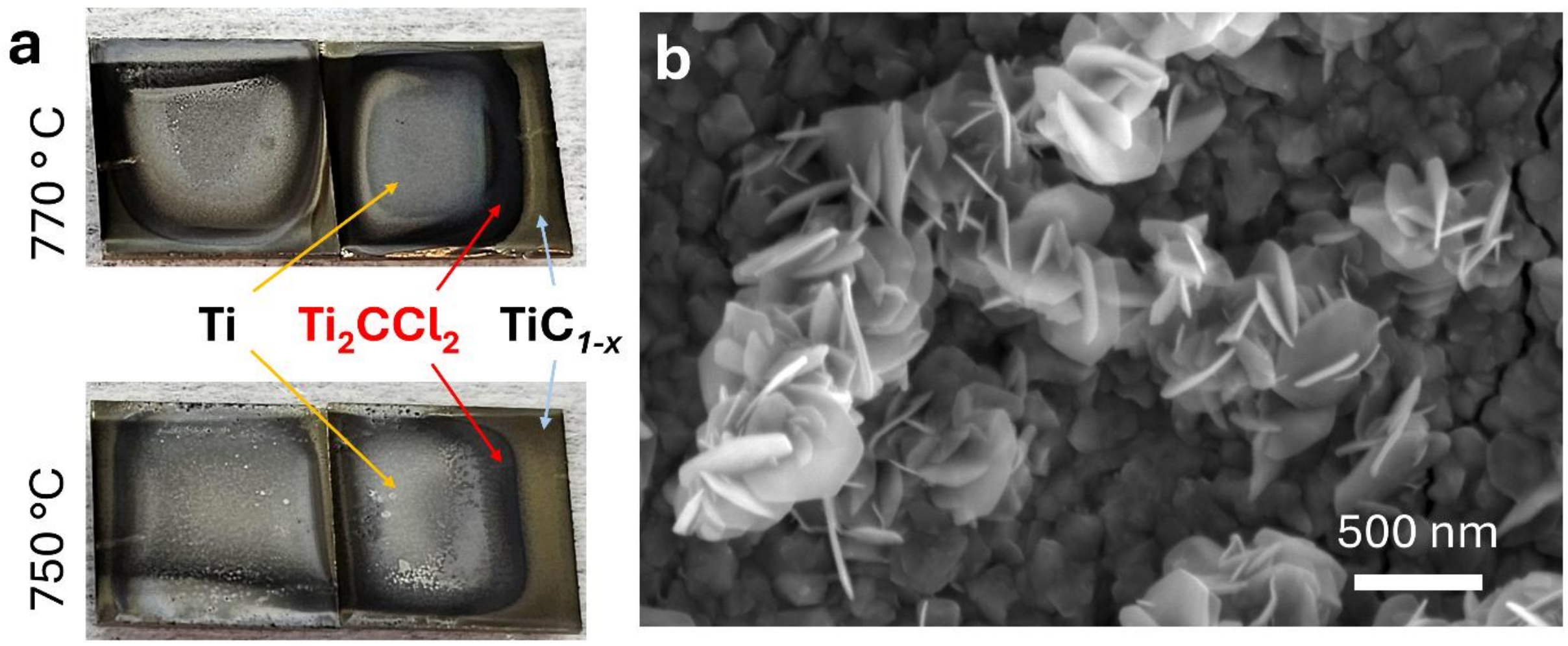

Figure S8. **(a)** Digital photographs of MXene samples on quartz substrates deposited at 750 and 770°C. The center area is grey colored (Ti), and the outer area is yellow (TiC_{1-x}, confirmed by Raman spectroscopy). At the boundary, small amounts of black particles are observed. **(b)** SEM revealed those as Ti_2CCl_2 MXene flakes on TiC_{1-x} grains. A much slower nucleation rate is possibly due to low $TiCl_2$ concentration or insufficient thermal energy. The local chemistry can be near the boundary condition of Ti_2CCl_2 and TiC_{1-x}. The growth rate is very slow, which is not effective for scalable production of Ti_2CCl_2. However, with extremely low precursor concentrations near the boundary condition, one may achieve continuous growth of large single flakes for electronic and quantum applications. The chlorinated titanium molecules ($TiCl_2$) prevent the substrate from being carburized, but the reaction to form Ti_2CCl_2 MXene is limited due to a low reaction temperature. At those lower temperatures, the balanced local chemistry might have been shifted from the center to the slightly outer area. The Ti area is where Ti is prevented from forming carbides, and the TiC_{1-x} area is carburized titanium, Ti_2CCl_2 MXene forms at the boundary, which is carburized and chlorinated titanium.

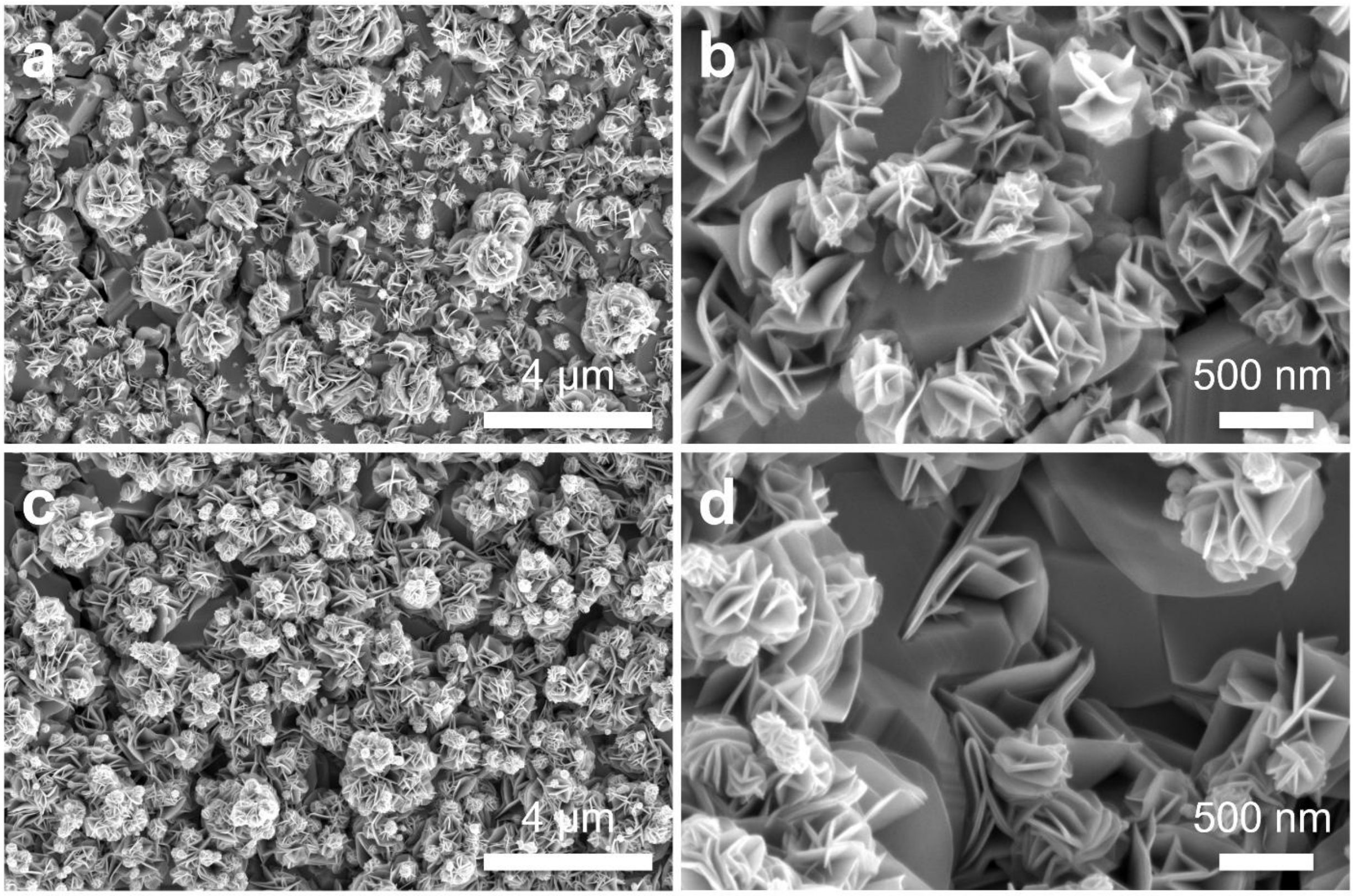

Figure S9. SEM images of Ti_2CCl_2 deposited on quartz substrates at relatively low temperatures for 2 h. Images show the top view of the spherulites assembly on a quartz substrate. Left and right columns are low- and high-magnification images, respectively: **a,b)** 800°C. **c,d)** 850°C. The darker and smoother area represents titanium grains formed on the quartz substrate prior to MXene deposition. The carburization and chlorination of titanium grains may produce MXene at the titanium grain boundaries as seen in the right part of panel (d).

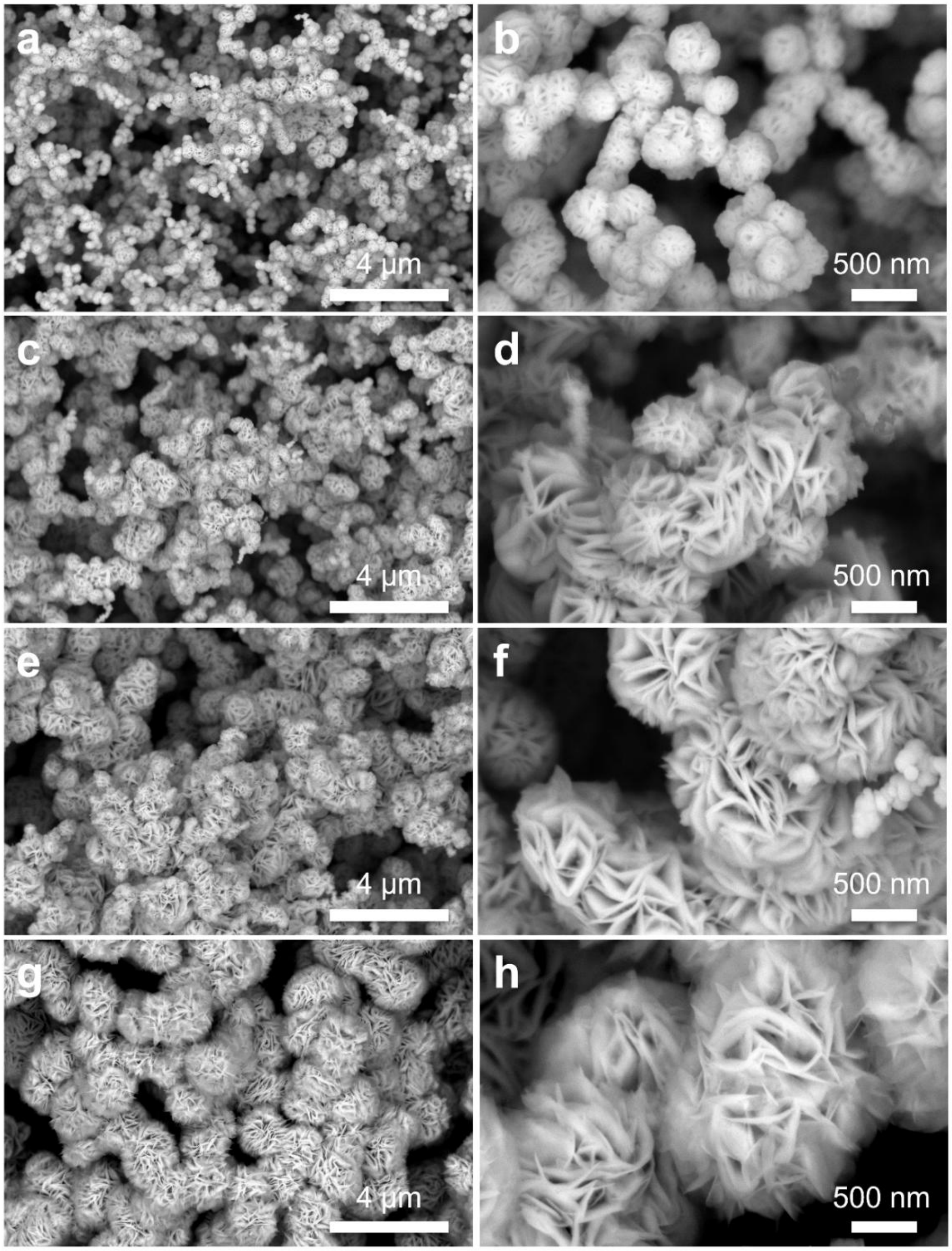

Figure S10. SEM images of Ti_2CCl_2 MXene spherulites deposited on quartz substrates at 950°C for reaction period from 1 h to 24 h. Images show the top view of the spherulites assembled on a quartz substrate. The left and right columns show low- and high-magnification images, respectively: **a,b)** 1h. **c,d)** 2h, **e,f)** 4h, **g,h)** 24h. Panel (h) is SEM image shown in Fig.2h captured by another detector simultaneously.

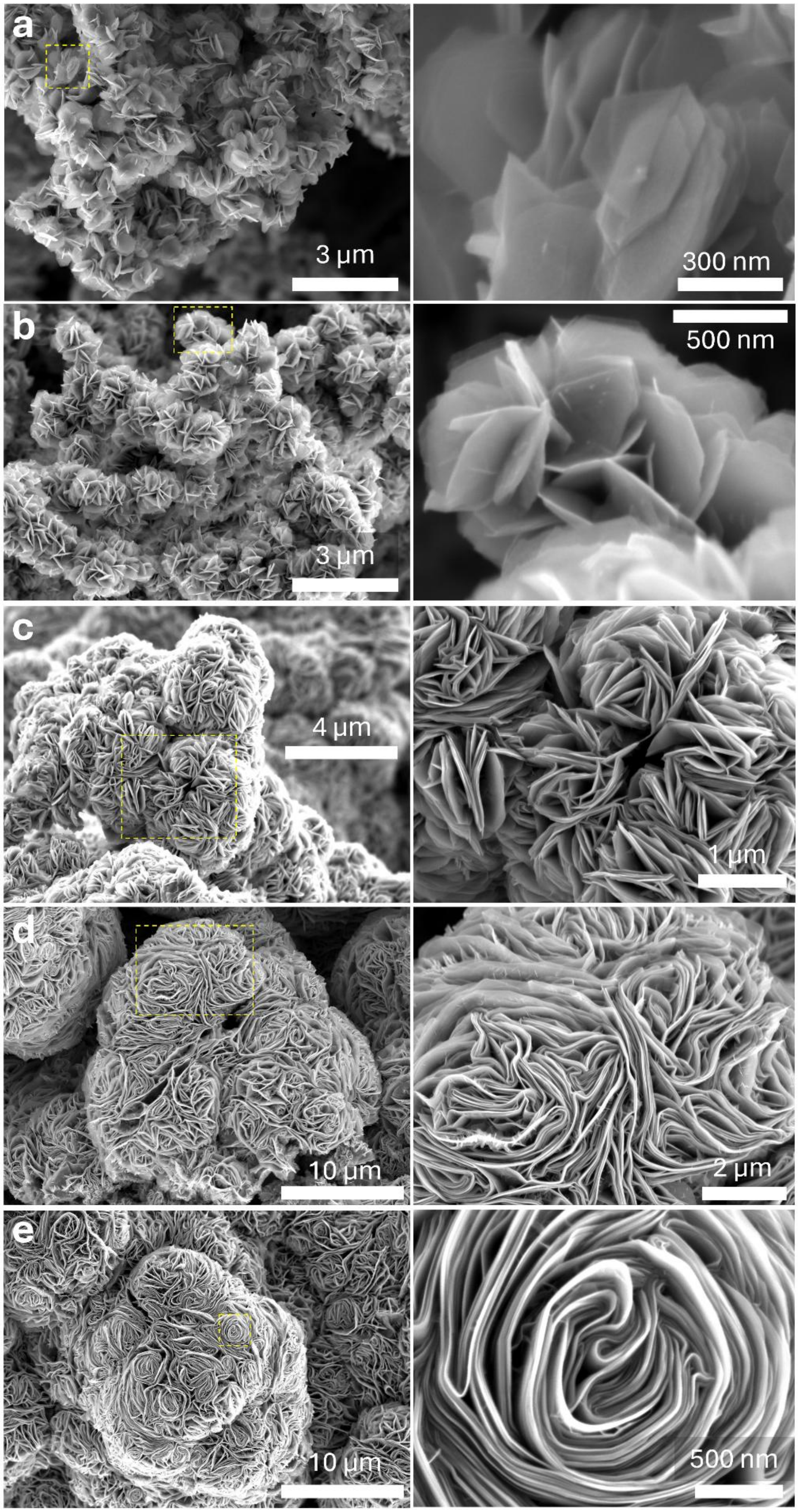

Figure S11. SEM images with low (left) and high magnification (right) of bulky Ti_2CCl_2 MXene grown within the reaction volume for 24 h at **(a-c)** 840°C and **(d,e)** 850°C. Images are presented in the order of cluster size, from the smallest in (a) to the largest in (e). Multiple different morphologies were obtained due to the different growth times for each particle cluster, as well as fluctuations in local chemistry and gas flow. These images represent the growth from small individual multilayer flakes to larger and denser cluster formations. The large particles present scrolled MXene sheets. Panel (e) corresponds to the lower- and the higher-magnification version of Fig.3a.

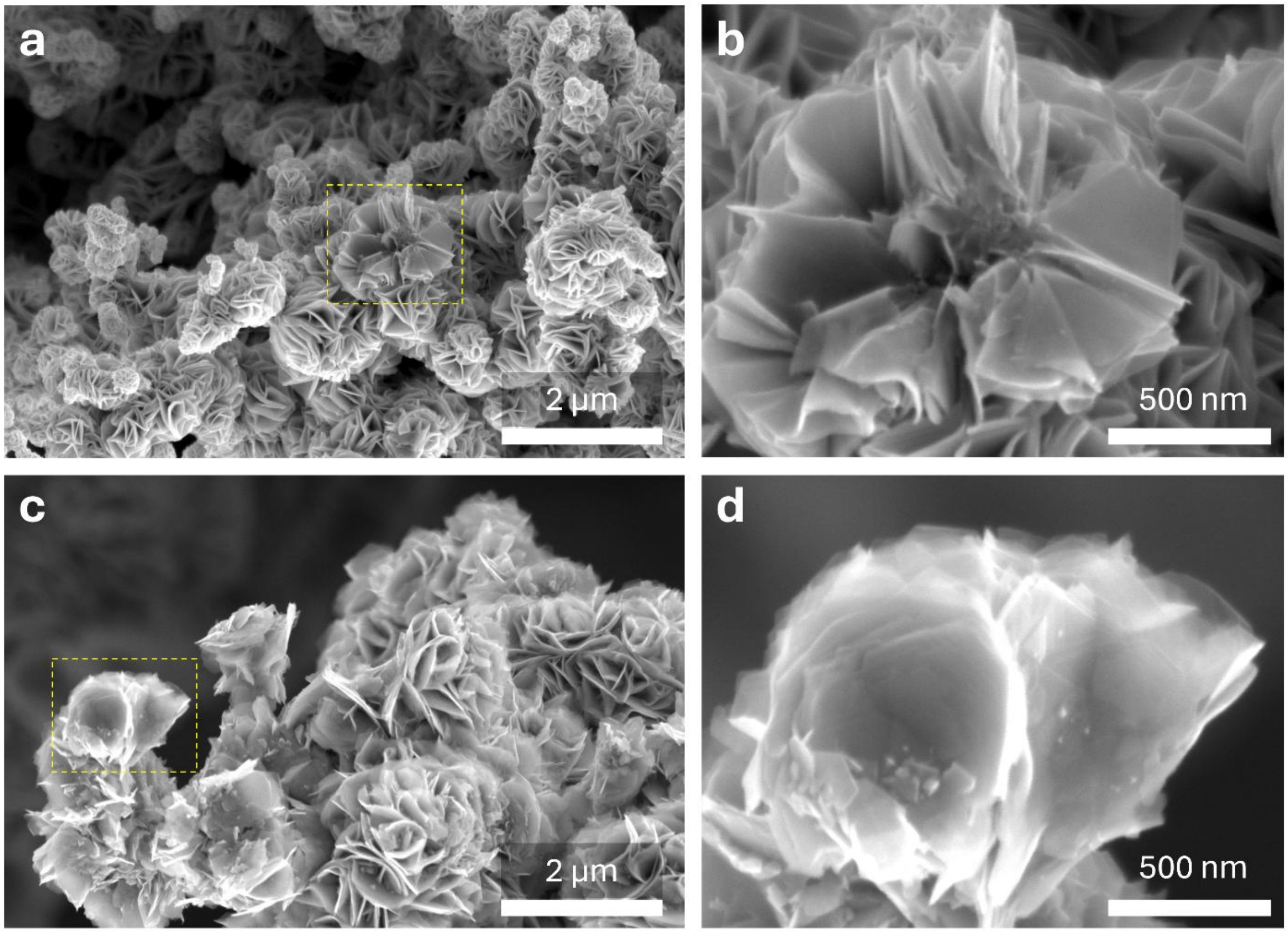

Figure S12. SEM images with low (left) and high magnification (right) of fractured MXene spherulites grown for (**a,b**) 2 h at 900°C, and (**c,d**) 24 h at 950°C. The fractured areas show that MXene flakes grow from the core to the outside, with lateral size equal to the radius of the spherulite. The adjunct spherulites may merge, leading to coarsening of the MXene flakes via decomposition, mass transport over short distances, and reassembly.

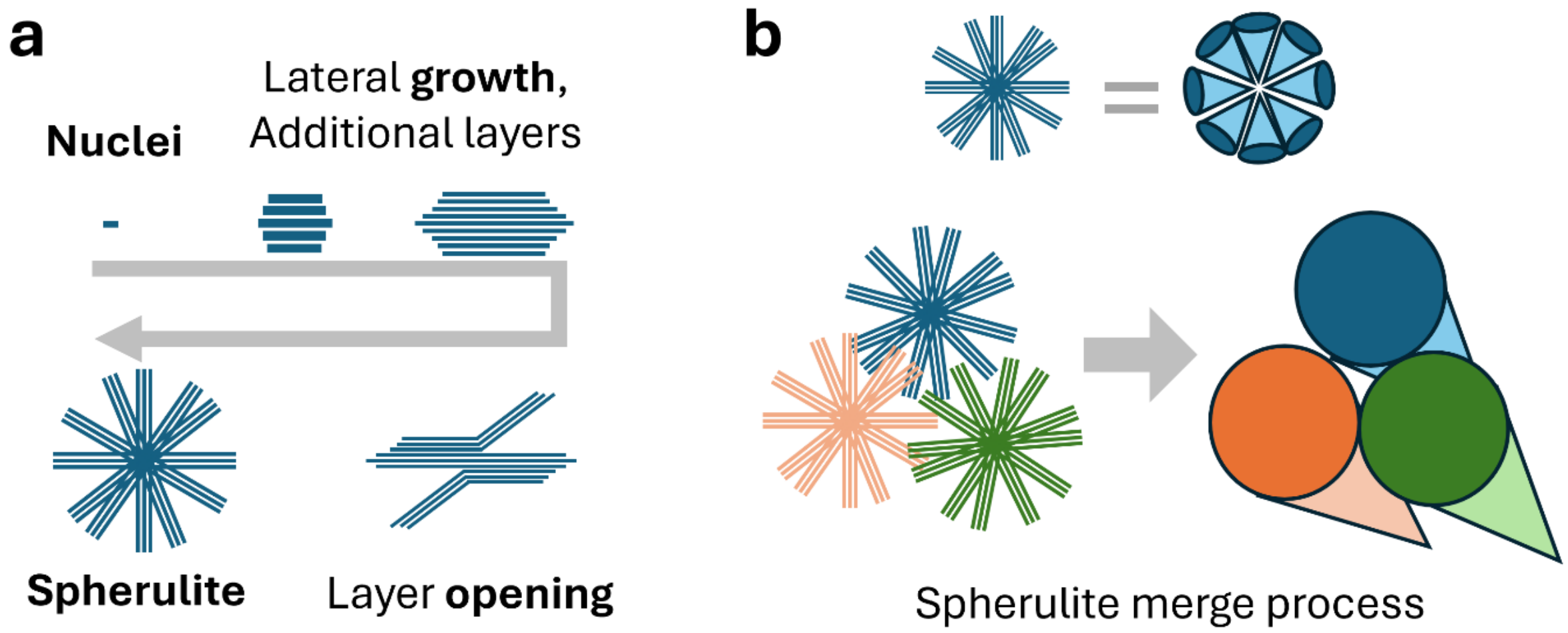

Figure S13. Schematic illustration of **(a)** MXene spherulites formation and **(b)** the merging process. Each small spherulites can become a part of the larger spherulite. The layer opening is attributed to the gas exchange during the MXene forming chemical reaction which should have been done in high growth rate.

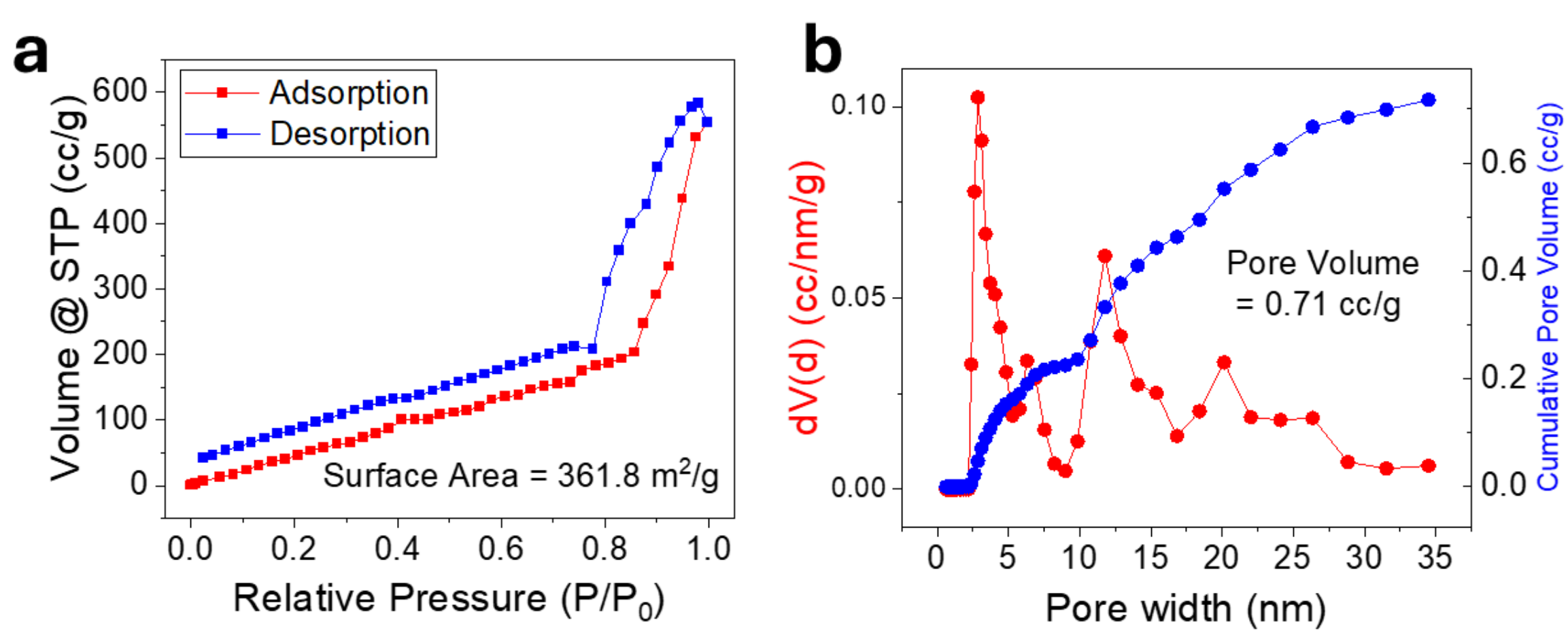

Figure S14. BET analysis of Ti_2CCl_2 MXene spherulites, formed after 2 h at 950°C. **(a)** Nitrogen isotherm at 77 K. **(b)** Pore size and volume distribution by QSDFT method.

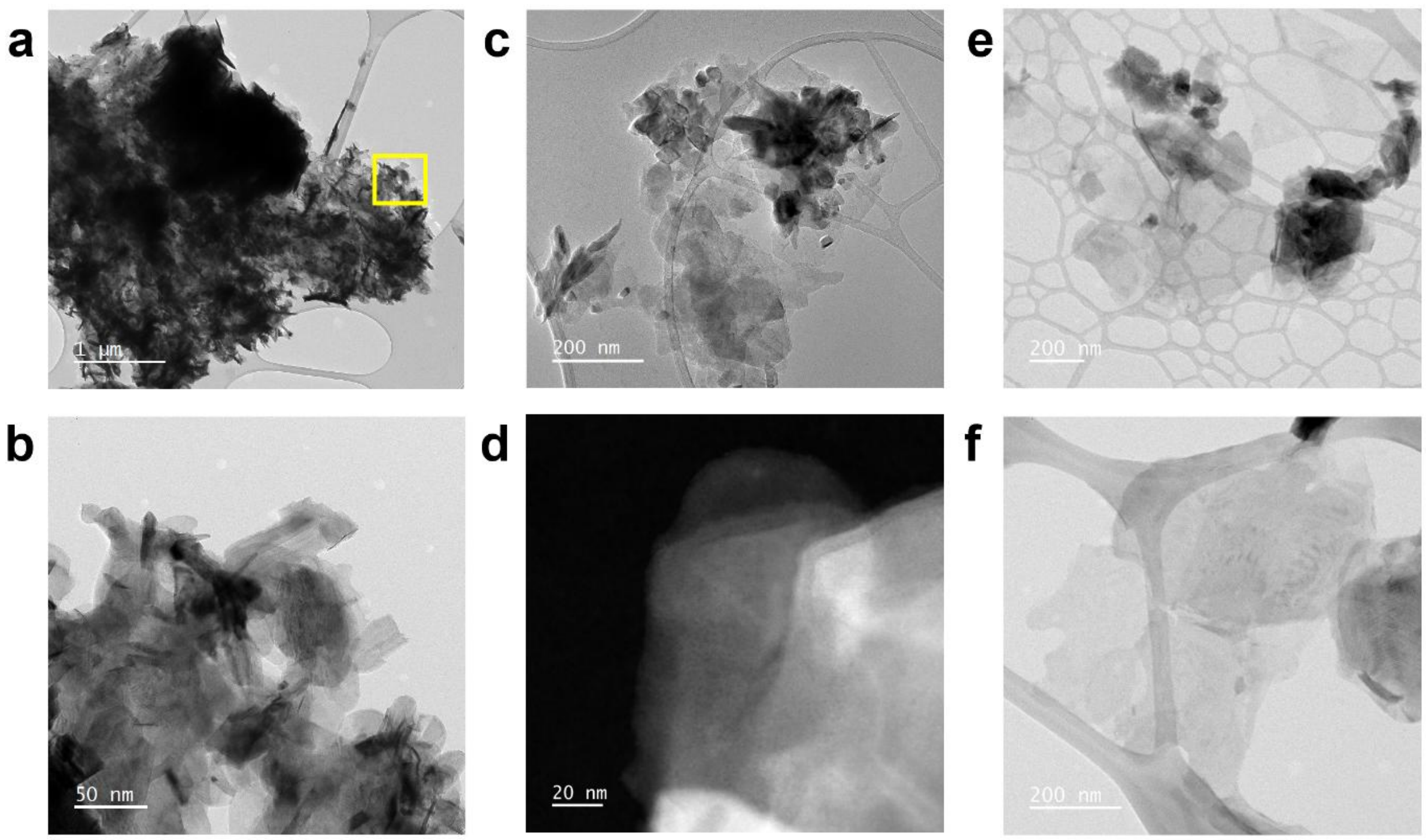

Figure S15. TEM images of Ti_2CCl_2 sample with different processing. **(a,b)** Sample prepared by simple ultrasonication in ethanol. [Few to multi-layers]. Yellow box in panel (a) indicates the corresponding high-magnification image in panel (b), **(c-f)** Samples prepared by involving Li^+ intercalation and delamination.; **(c-d)** LiCl intercalation in organic solvent used in this study, following Ref.[29] resulted in few to monolayer. **(e,f)** n-butyllithium intercalation show few to monolayer. (a,c,e) low-, and (b,d,f) high-magnification images.
